# *Ehretia* genus: a comprehensive review of its botany, ethnomedicinal values, phytochemistry, pharmacology, toxicology and clinical studies

**DOI:** 10.3389/fphar.2025.1526359

**Published:** 2025-02-21

**Authors:** Sai Jiang, Mengyun Wang, Amanpreet Kaur, Lin Jiang, Yuan Cai, Jiangyi Luo, Minxi Li, Hongxing Wang, Dan Wan, Yanmei Peng

**Affiliations:** ^1^ Institute of Innovative Traditional Chinese Medications, Hunan Academy of Chinese Medicine, Changsha, China; ^2^ TCM and Ethnomedicine Innovation and Development International Laboratory, Innovative Material Medical Research Institute, School of Pharmacy, Hunan University of Chinese Medicine, Changsha, China; ^3^ Department of Chemistry, School of Sciences, IFTM University, Moradabad, Uttar Pradesh, India; ^4^ Forestry Bureau of Hengnan County, Hengyang, China

**Keywords:** *Ehretia genus*, traditional uses, phytochemistry, pharmacology, clinical studies

## Abstract

**Background:**

The *Ehretia* genus, comprising 66 species in the Boraginaceae family, has a history of ethnomedicinal use for various ailments. This review focuses on the botany, traditional uses, phytochemistry, pharmacology, toxicology, clinical studies, cultivation, and commercial potential of the *Ehretia* genus, with the goal of enhancing current research and applications.

**Methods:**

Literatures related to *Ehretia* species were compiled using keywords such as “*Ehretia*,” “traditional use,” “chemical constituents,” and “bioactivity” from scientific databases, including “China Knowledge Resource Integrated Databases (CNKI),” “Flora of China,” “Google Scholar,” “Hunan Library,” “Plants of the World Online,” and “Web of Science” and so on.

**Results:**

From 1980 to August 2024, only 101 compounds have been identified within this genus, primarily consisting of flavonoids, phenylpropanoids, phenolics, benzoquinones, triterpenoids, and fatty acids, with phenylpropanoids being the main components. Extracts and compounds from *Ehretia* species exhibited various bioactivities, including antioxidant, hepatoprotective, analgesic, anti-inflammatory, antibacterial, and anticancer effects, etc.

**Conclusion:**

Research on the *Ehretia* genus is limited, with many species remaining underexamined in terms of phytochemistry and pharmacology. Few active compounds have been isolated and assessed for biological activities, and there is a lack of investigation into their mechanisms of action. Despite its documented uses, *Ehretia* species remains less explored scientifically than other Boraginaceae genera, presenting significant research opportunities. Further comprehensive studies are necessary to deepen our understanding of this diverse genus and validate its therapeutic potential.

## Graphical Abstract

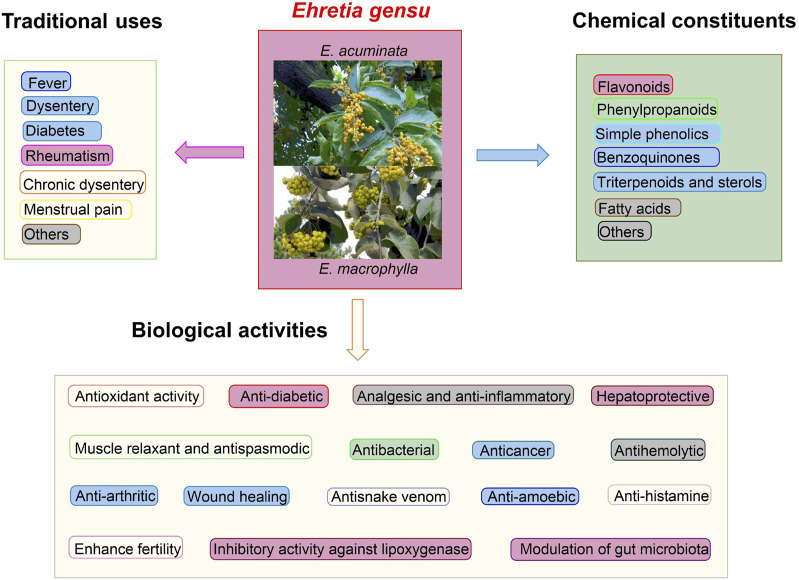

## 1 Introduction

The *Ehretia* genus, comprising 66 accepted species, belongs to the Boraginaceae family and is primarily distributed across tropical Asia, Africa, Australia, North and South America (http://www.plantsoftheworldonline.org). These plants exhibit wide adaptability and strong stress resistance, thriving in warm, moist environments with deep, fertile, well-drained soil (pH 5.5–6.5) and an annual average temperature above 10°C. *Ehretia* species grow in various habitats, from tropical forests to dry regions, showcasing resilience compared to less adaptive genera like Myosotis (Mandal and Joshi, 2014; Retief and Van Wyk, 2001; Shukla and Kaur, 2018). The genus includes only tree or shrub species (http://www.iplant.cn/). Various parts of the *Ehretia* plants-roots, leaves, flowers, barks, fruits, and heartwoods-are used in herbal medicine and consumed as food (Li et al., 2010a; Lim et al., 2023; Hadjichambis et al., 2008; Zara et al., 2012). In folklore, *Ehretia* species were employed as ethnomedicine to treat a variety of cough, inflammation, swellings and syphilis, et al. (Iqbal et al., 2005). Notably, some species such as *Ehretia corylifolia*, *E. longiflora*, *E. macrophylla*, *E. thyrsiflora*, and *E. tsangii* are recognized for their antipyretic and detoxifying properties in traditional Chinese medicine (Zeng and Zeng, 1994). The bitter tea known as “Kudingcha” in Chinese has historically been made locally using the leaves of *E. thyrsiflora* (He and Liu, 1992). “Tsaang gubat” also known as *E. microphylla*, is presently included in the Philippine National Formulary and is recognised by the Department of Health as one of the top therapeutic plants recommended in the nation (Legaspi and Bagaoisan, 2020).

According to literatures, more than 100 compounds have been identified from *Ehretia* genus. These compounds including flavonoids, phenylpropanoids, simple phenolics, benzoquinones, triterpenoids, fatty acids and so on (Chien et al., 2012; Le et al., 2021; Li et al., 2009; Yamamura et al., 1995; Yoshikawa et al., 1995). The extracts and compounds of *Ehretia* species exhibited a range of bioactivities, such as antioxidant, anti-diabetic, hepatoprotective, analgesic, anti-inflammatory, antibacterial, anticancer, antihemolytic, anti-arthritic, wound healing effects, etc (Harne et al., 2021; Jan et al., 2023; Kaur et al., 2019; Kaur et al., 2022; Memon et al., 2022; Panja et al., 2020; Rangnathrao and Shanmugasundaram, 2019; Waheed et al., 2019).

To date, only two review articles focus on the genus *Ehretia*, with the most recent one occurring 6 years ago (Li et al., 2010a; Shukla and Kaur, 2018). This review aims to provide a comprehensive overview of the botany, ethnomedicinal values, phytochemistry, pharmacology, toxicology, clinical studies, cultivation, and commercial value of the genus *Ehretia* (Mitsi and Echeverría, 2024). Due to the genus’s diversity, only species with proven medicinal characteristics will be highlighted. Additionally, this review addresses the limitations of current research and suggests directions for future studies, offering insights to guide upcoming research on *Ehretia* species.

## 2 Ethnomedicinal uses of genus *Ehretia*

**FIGURE 1:**
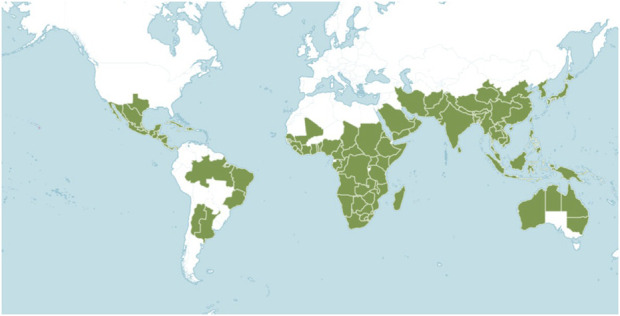
Geographical distribution of *Ehretia* species plants in the world (Cite from http://www.plantsoftheworldonline.org).

The Flora Reipubae Popularis Sinicae categorizes all plants in the genus *Ehretia* as either trees or shrubs (Table 1) (Editorial Committee of the Flora of China, 1985). This genus is predominantly found in southern Asia, China, Africa, North and South America (Figure 1). In China, twelve species and one variant are identified, primarily in provinces south of the Yangtze River, with additional occurrences in southern Gansu, Henan, Shaanxi, and Qinghai. It is only known that 12 species of the genus are utilized in traditional medicine (Table 2). Rich folklore medical applications for *Ehretia* plants include treatments for syphilis, eczema, stomach disorders, cough, diarrhea, and chest pains (Arenas et al., 2013; Mncwangi et al., 2012; Torane et al., 2011; Waheed et al., 2019). The *Ehretia* genus also contributes significantly to forest ecosystems as a source of food for birds and insects due to its fleshy drupes and nectar-rich flowers. Unlike invasive or fast-spreading members of Boraginaceae (e.g., *Heliotropium indicum*), *Ehretia* species are generally regarded as ecologically harmonious, adding to their conservation importance.

**TABLE 1:** Morphological features of *Ehretia* genus (Flora of China).

Part	Features
Leaf	Leaves petiolate, entire or serrate at margin
Flower	Inflorescences corymbose or paniculate-cymose. Calyx 5- lobed. Corolla white or pale yellow, tubular or tubular-campanulate, rarely funnelform, 5-lobed; lobes spreading or reflexed. Filaments usually exserted; anthers ovate to oblong or linear. Ovary ovoid, 2-loculed, each locule with 2 ovules. Style terminal, 2-cleft; stigmas 2, capitate or elongated
Fruit	Drupes yellow, orange, black, or pale red, subglobose, glabrous, endocarp divided at maturity into 2 2-seeded or 4 1-seeded pyrenes

**TABLE 2:** Record of the traditional uses of some *Ehretia* species (http://www.plantsoftheworldonline.org).

Accepted name	Synonym name	Common name	Traditional use	References
*Ehretia acuminata* R.Br	*Ehretia thyrsiflora* var. latifolia Nakai *Ehretia serrata* Roxb. *Ehretia ovalifolia* Hassk	Kudingcha (China) Puna (Pakistan); Koda; Pudila; Nara	To cure fever, sores on tongue, dysentery	Choudhury et al. (2012) and Wang and Huang (2005)
*Ehretia anacua* (Terán and Berland.) I.M.Johnst	*Gaza anacua* Terán and Berland		To treat diabetes	Neuwinger (2000)
*Ehretia asperula* Zoll. and Moritzi	*Ehretia hanceana* Hemsl		To cure hepatitis, liver cirrhosis, and cancer	Hoang et al. (2021) and Le et al. (2021)
*Ehretia cymosa* Thonn	*Ehretia thonningiana* Exell	Game (Amharic); Hulaga; Ulaga; Garmi (Afan Oromo)	To cure rheumatism, headache, measles and febrile illness	Alemayehu et al. (2015), Ashagrie et al. (2023), Fassil and Gashaw (2019), and Ogundajo et al. (2016)
*Ehretia dicksonii* Hance (*Ehretia macrophylla* Wall.)		Cukangsu (China)	To cure sphagitis, amygdalitis, and coughs	Deng et al. (2020), Liu (2003), and Xu et al. (2022)
*Ehretia aspera* Willd	*Ehretia laevis* Roxb *Bourreria aspera* (Willd.) G.Don	Chamror (Punjab); Kuptaa; Datarangi (Maharashtra)	To cure headaches, and ulcers, anthelmintic, diuretic, demulcent, expectorant, astringent and nosh	Joshi and Wagh (2019)
*Ehretia microphylla* Lam	*Ehretia buxifolia* var. microphylla (Lam.) DC *Carmona microphylla* (Lam.) G.Don		To treat bloody discharge and dysentery, leprosy, eczema due to venereal diseases, chronic dysentery, infertility and toxic diarrhea in children	Ali et al. (2019), Kudera et al. (2021), and Yuvaraja et al. (2021)
*Ehretia obtusifolia* Hochst. ex ADC	*Ehretia aspera* var. obtusifolia (Hochst. ex A.DC.) Parmar		To cure sore throat, teething pains in infants, menstrual pain, abdominal pains and infertility in women	Jan et al. (2023)
*Ehretia philippinensis* ADC			To treat certain inflammatory processes	Simpol et al. (1994)
*Ehretia tinifolia* L	*Ehretia campestris* Salisb		To treat urinary track disorder by reducing uric acid	Lim et al. (2023) and Pío-León et al. (2012a)
*Ehretia rigida* (Thunb.) Druce	*Capraria rigida* Thunb *Freylinia rigida* (Thunb.) G.Don	Cape lilac	To treat infertility, headache, abdominapains, chest pains, pain, skin cuts, sprained joints, newborn baby infections	Maroyi (2023)

**FIGURE 2:**
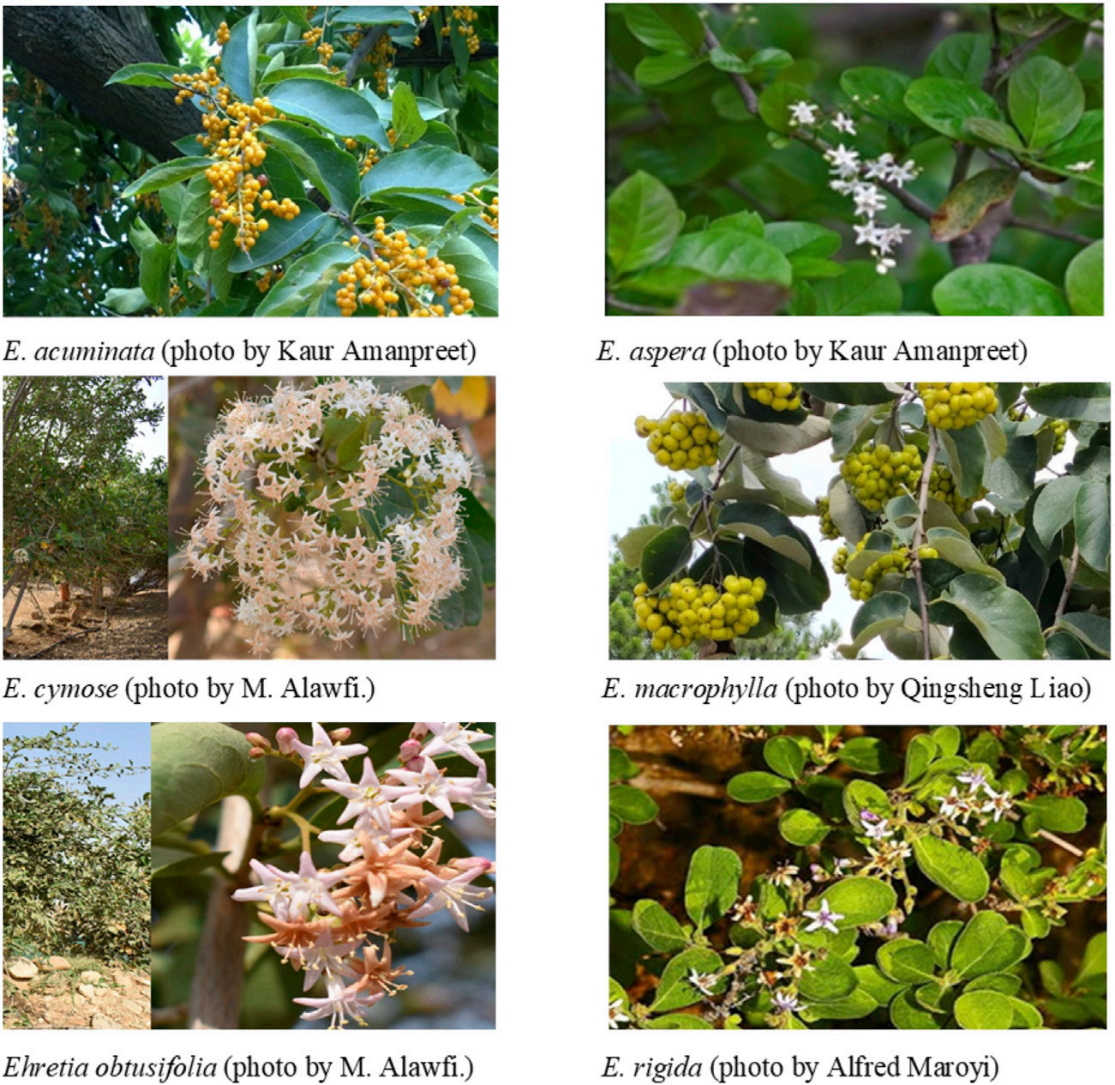
The pictures of fruits, leaves and flowers of some *Ehretia* species plants.

Among the earliest species in the *Ehretia* genus is *E. acuminata*, which has been traditionally used to treat fever, tongue sores, diarrhea, and other ailments in China and India (Choudhury et al., 2012; Shukla et al., 2019b; Wang and Huang, 2005). The leaves of *E. thyrsiflora* (syn. *E. acuminata*) serve as one of the sources for Kudingcha, a bitter tea popular in southern China (Li et al., 2009). In Nigeria, folk medicine utilizes the leaves of *E. anacua* to cure diabetes (Abimbola et al., 2021). *E. asperula*, endemic to northern Vietnam, has a history of use in treating cancer, liver cirrhosis, and hepatitis (Hoang et al., 2021; Le et al., 2021). The small tree *E. cymose* is predominantly found in the secondary jungles and savannas of Africa. Its fruits are black, ovoid to spherical, measuring 2–6 mm in length, and the leaves are oval in shape (Sarkodie et al., 2015). In several parts of Ethiopia, the leaves of *E. cymose* are traditionally employed to treat feverish illnesses, rheumatism, headaches, and measles (Figure 2) (Alemayehu et al., 2015; Ashagrie et al., 2023; Fassil and Gashaw, 2019; Ogundajo et al., 2016).

Folk medicine for alleviating swelling often utilizes the bark of *E. dicksonii* (Xu et al., 2022). For respiratory conditions such as sphagitis, amygdalitis, and coughs, the fruit of *E. macrophylla*-identified as the same species as *E. dicksonii*-along with herbal tea, serves as traditional remedies (Deng et al., 2020; Liu, 2003). *E. laevis*, a deciduous shrub native to tropical regions of Asia and Australia, is recognized for its significant therapeutic value in traditional medicine (Velappan and Thangaraj, 2014). In Uttarakhand and other sub-Himalayan regions of India, the herbal medicine is used to treat jaundice (Rangnathrao and Shanmugasundaram, 2019). Its utilization can be traced back to ancient medical systems, including Ayurveda and Siddha (Sharma et al., 2021). The plant uses its inner bark for sustenance and nutrition. This plant’s leaves can be used to cure headaches, skin conditions, and ulcers. This plant’s fruit is frequently used to treat lung and spleen ailments, urinary tract issues, astringent, deworming, diuretic, analgesic, and expectorant conditions (Joshi and Wagh, 2019).

*Carmona retusa* (syn. *E. microphylla*) is a striking shrub reaching heights of 1.5–4.0 m, characterized by its glossy, coarse, dark green leaves. In addition to medicinal uses, certain species like *E. microphylla* are also cultivated for ornamental purposes and bonsai (Aarthi et al., 2014; Mageswari and Karpagam, 2015; Mageswari et al., 2012). *E. microphylla* is particularly significant in the Philippines and India, especially within the Ayurvedic and Siddha medical traditions (Chopra et al., 1956; Selvanayagam et al., 1996). A leaf infusion serves as a tea alternative and cough suppressant for conditions involving bloody discharge and diarrhea (Ali et al., 2019; Yuvaraja et al., 2021). In Siddha Materia Medica, it is used to treat leprosy, sexually transmitted infection-related dermatitis, chronic dysentery, infertility, and toxic diarrhea in children (Kudera et al., 2021). Additionally, the root acts as an antidote for vegetable poisoning and treats syphilis and cachexia (Aarthi et al., 2014).

In Zimbabwe, various compounds from *E. obtusefolia* are utilized to cure infertility in women, menstrual cramps, stomach spasms, sore throats, and children’s teething pain (Jan et al., 2023). *E. philippinensis*, endemic to low- and medium-altitude forests in the Philippines, is known for its stem bark and leaves that aid in treating certain inflammatory processes (Simpol et al., 1994). The small deciduous tree or shrub *E. rigida*, also known as the “puzzle bush,” has its stems used medicinally by the Zulu people, and its fruit is edible (Steyn, 1998).

*E. tinifolia*, an evergreen tree native to Mexico and the United States, can grow up to 25 m. Its small, round, fragrant yellow fruit is commonly consumed, while the bark aids wound recovery, and the flowers and leaves can alleviate symptoms of bloody vomiting (Hadjichambis et al., 2008; Pío-León et al., 2012b; Lim et al., 2023; Pío-León et al., 2012a). In conclusion, various *Ehretia* species are primarily employed for diverse ailments in Asian countries, indicating a substantial potential for bioactive compounds. Consequently, these species warrant further biological and chemical investigation.

## 3 Phytochemistry

**FIGURE 3:**
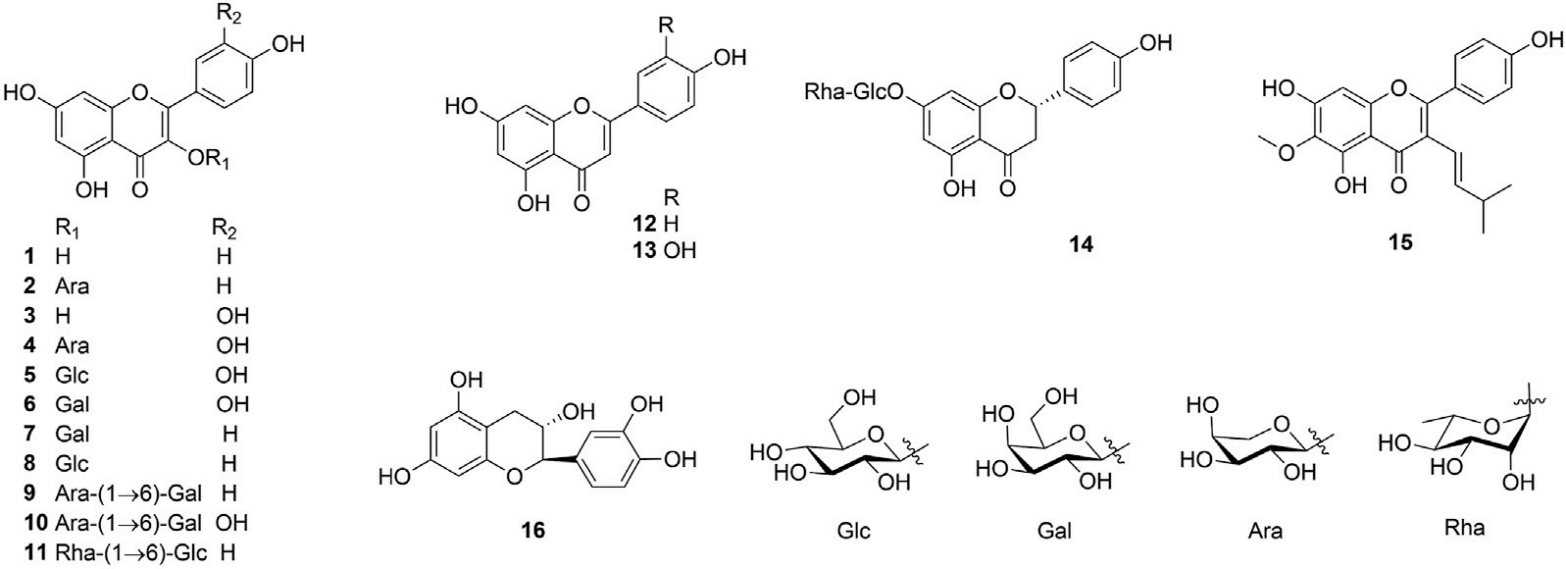
The structure of flavonoids from *Ehretia* species.

**FIGURE 4:**
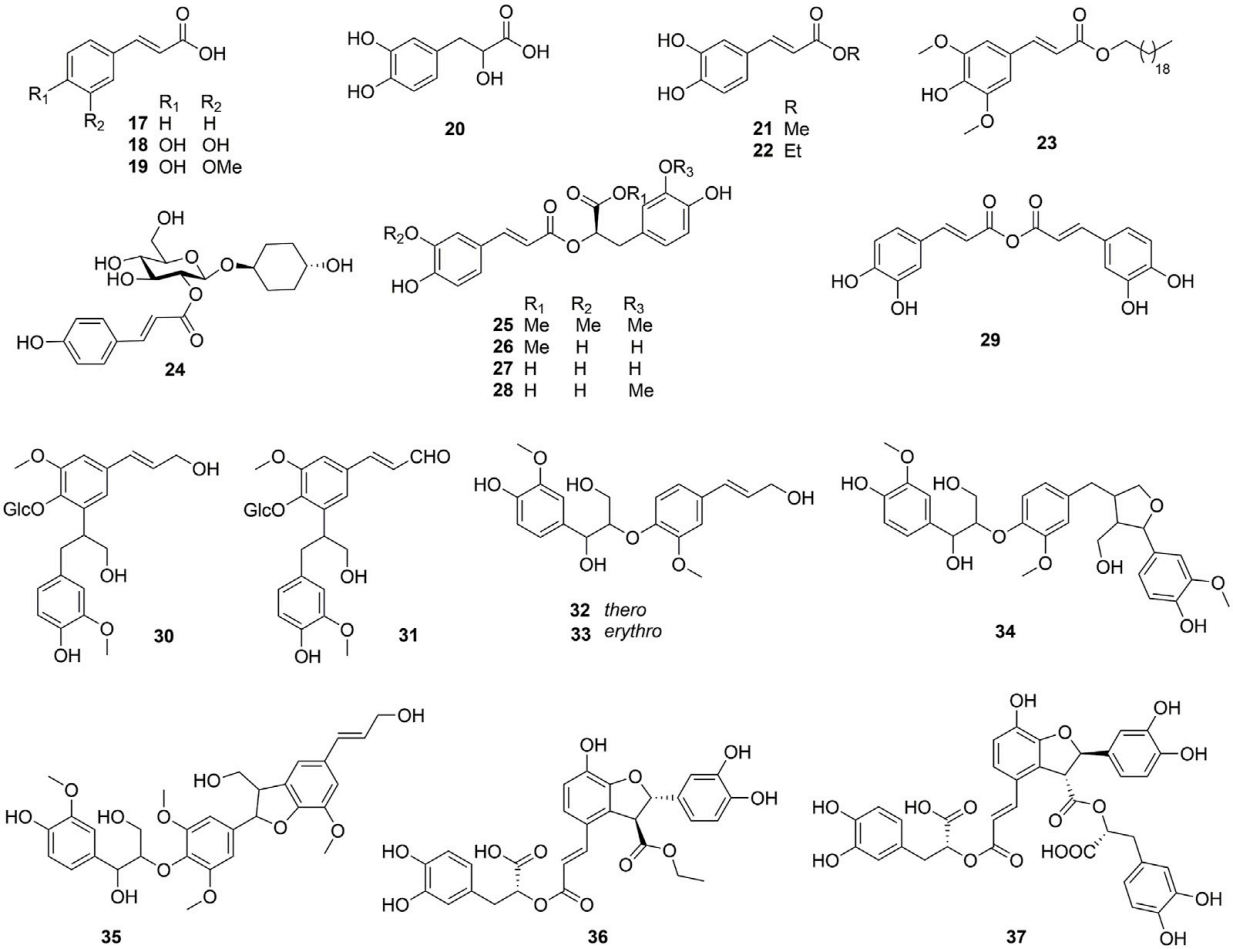
The structure of phenylpropanoids from *Ehretia* species.

**FIGURE 5:**
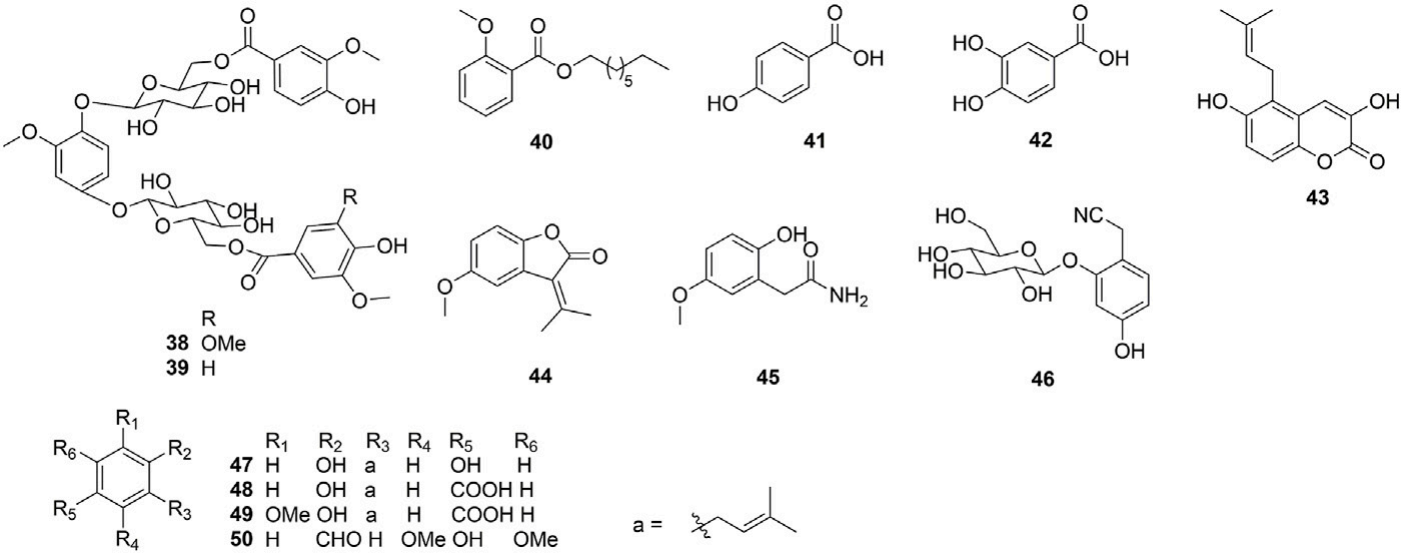
The structure of simple phenolics from *Ehretia* species.

**FIGURE 6:**
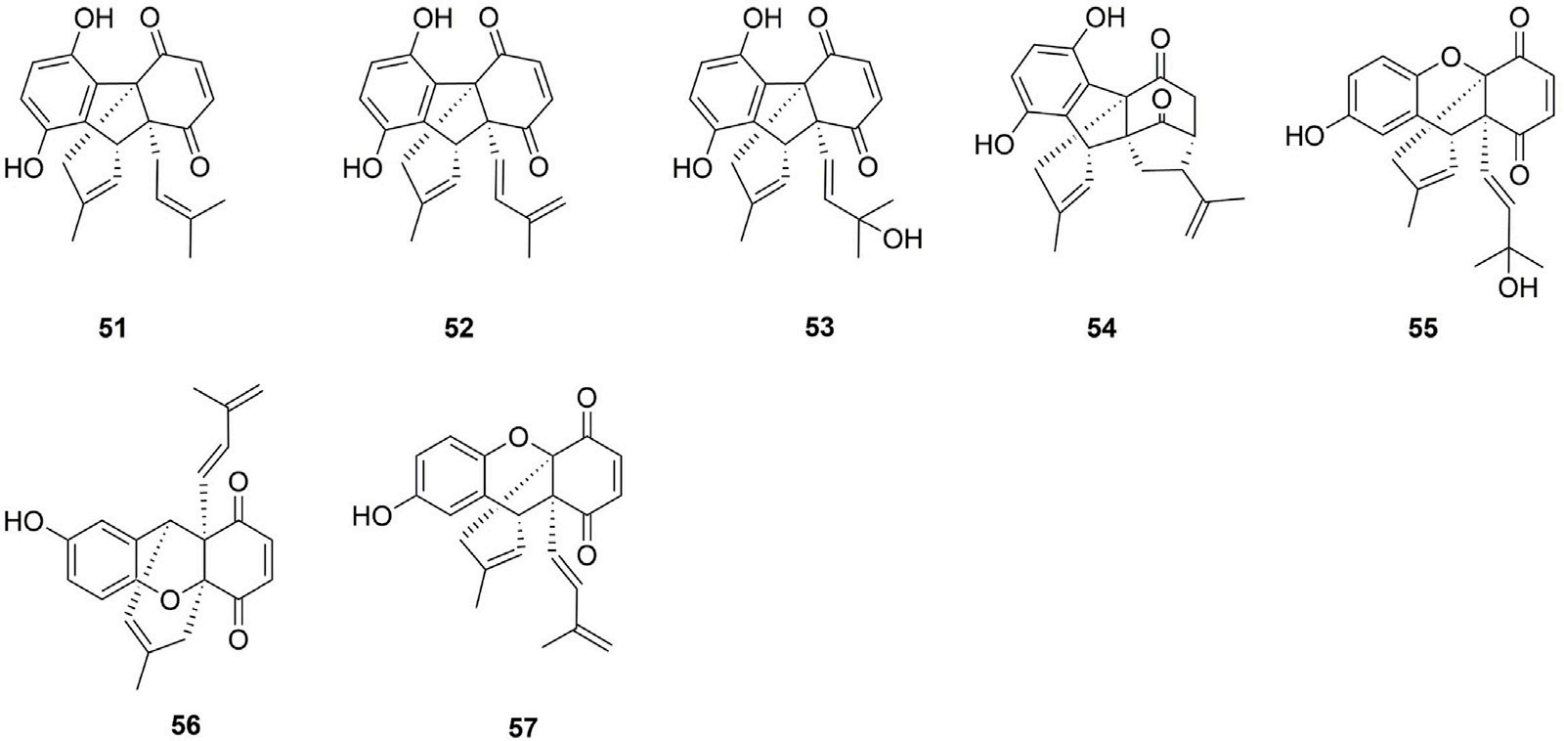
The structure of benzoquinones from *Ehretia* species.

**FIGURE 7:**
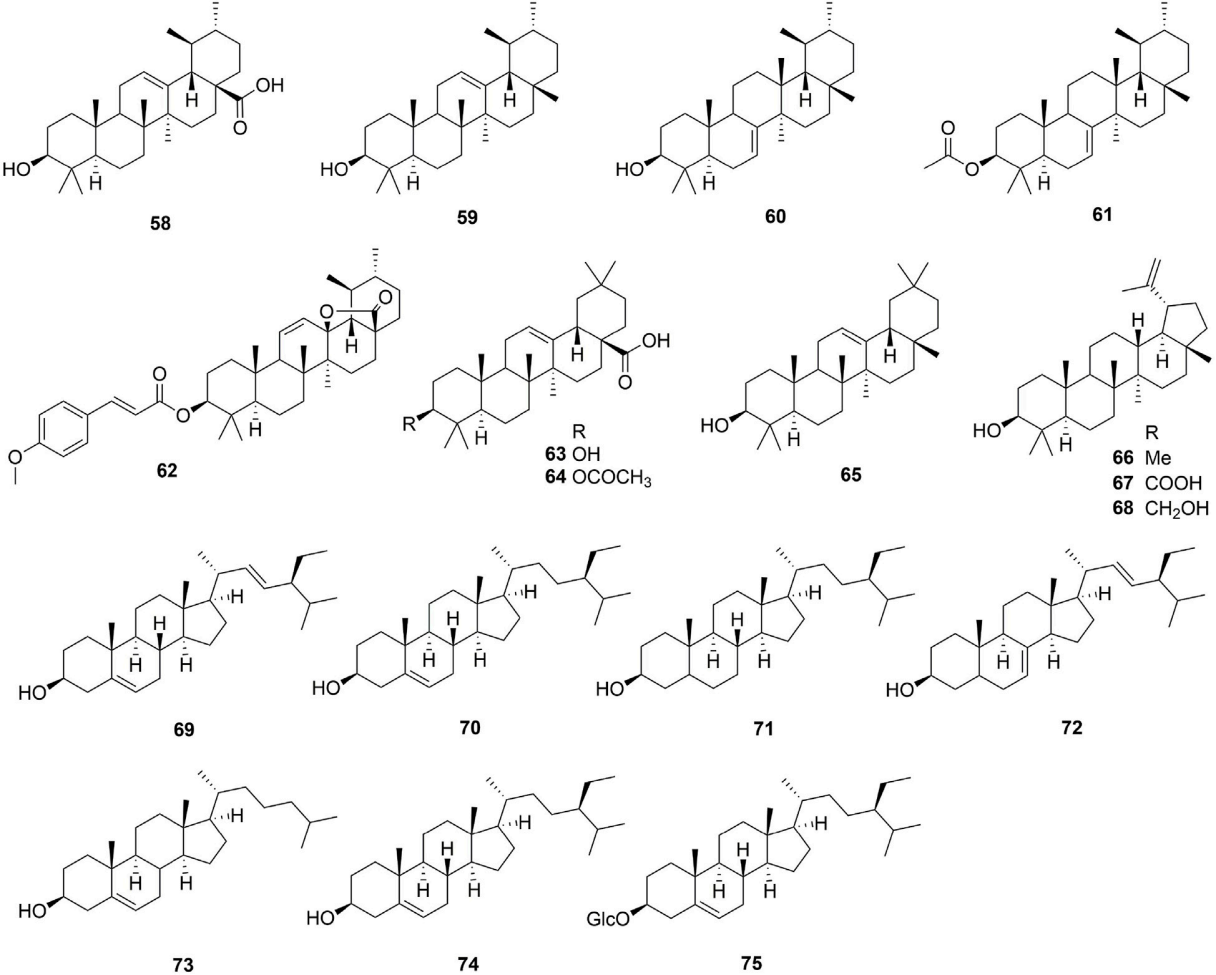
The structure of triterpenoids and sterols from *Ehretia* species.

**FIGURE 8:**
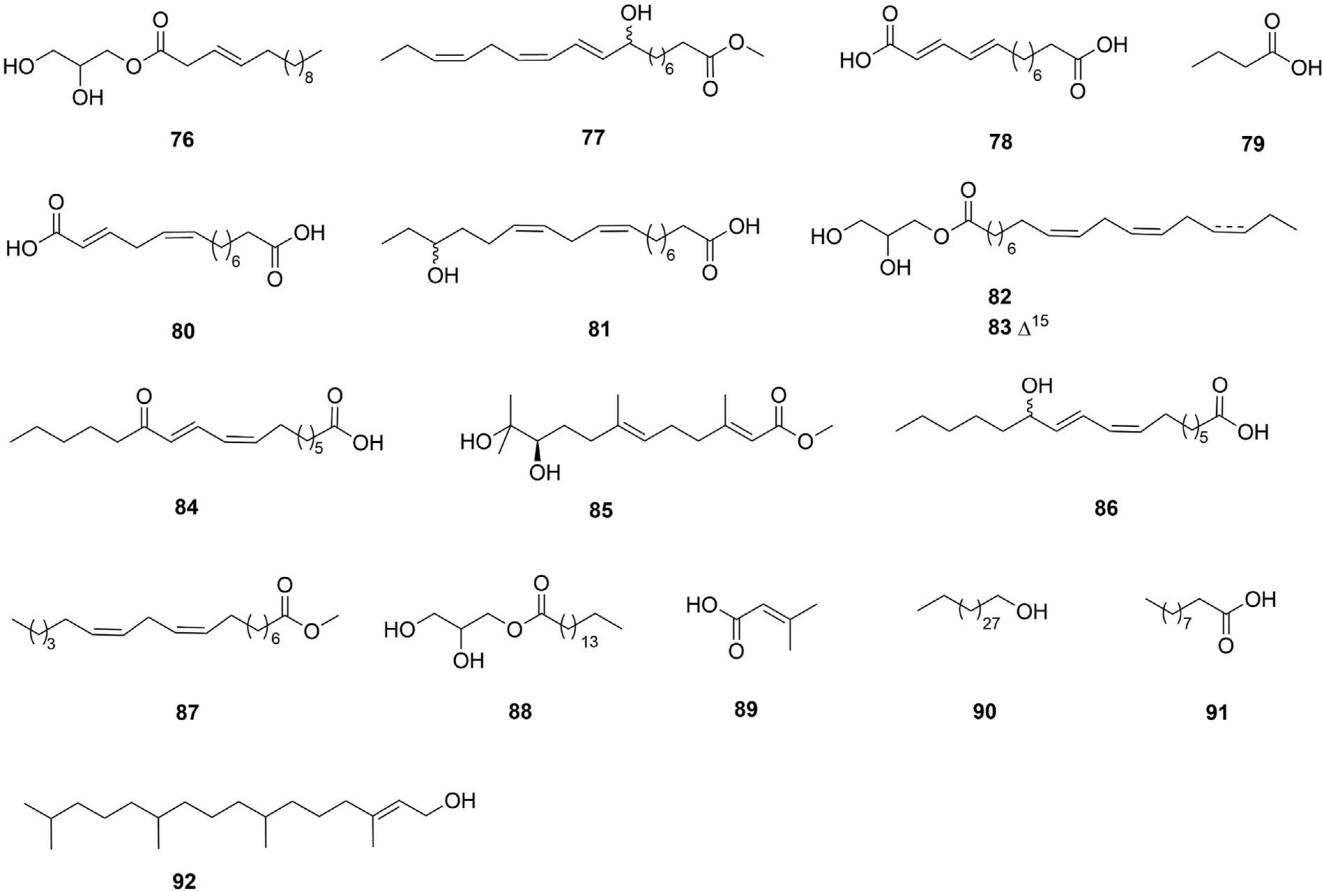
The structure of fatty acids from *Ehretia* species.

**FIGURE 9:**
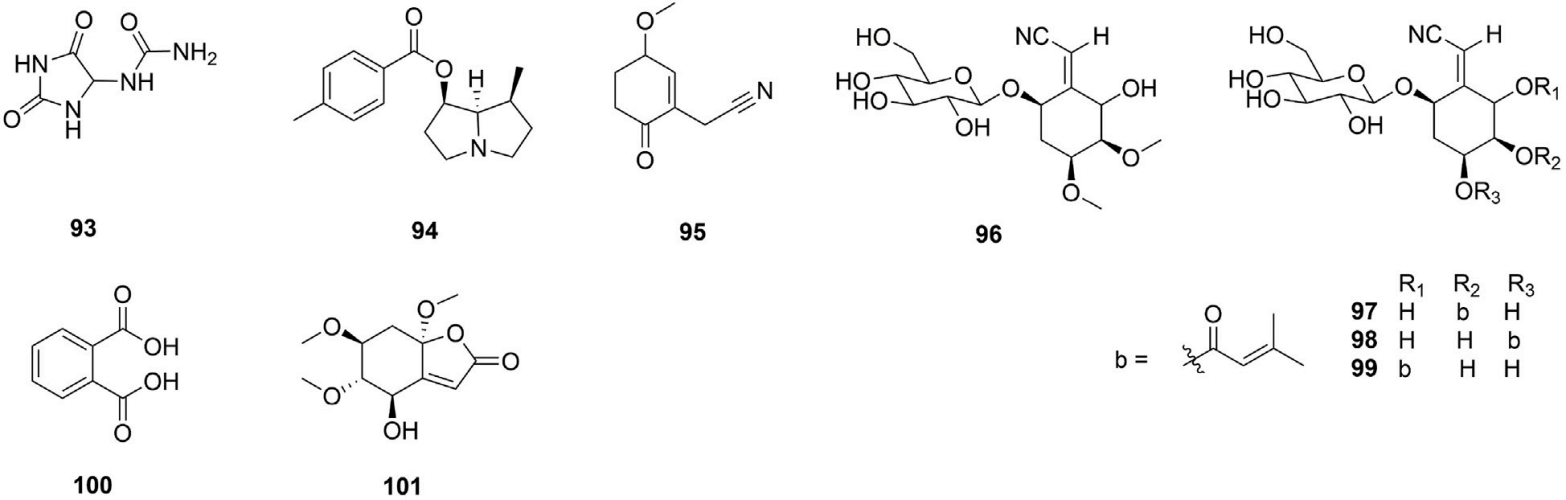
The structure of other compounds from *Ehretia* species.

One hundred and one compounds were identified from *Ehretia* genus, including flavonoids, phenypropanoids, simple phenolics, benzoquinones, triterpenoids, sterols, fatty acids and other compounds (Figures 3–9). These compounds have been identified in a variety of *Ehretia* species, including the fruit, roots, leaves, and bark (Table 3). Certain chemicals exhibited numerous bioactivities both *in vitro* and *in vivo*.

**TABLE 3:** Compounds from different parts of *Ehretia* genus by different identification methods.

No.	Compounds	Species	Parts used	Identification methods	Ref.
Flavonoids					
1	Kampferol	*E*. *thyrsiflora*	Leaves	1D NMR; ESIMS	Li et al. (2008)
2	Kampferol 3-*O*-*α*-D-arabinoside	*E*. *thyrsiflora*	Leaves	1D NMR; ESIMS	Li et al. (2008)
3	Querceti	*E*. *thyrsiflora* *E. macrophylla*	Leaves Fruit	1D NMR; ESIMS HPLC	Li et al. (2008) He, (2018)
4	Querceti 3-*O*-*α*-D-arabinoside	*E*. *thyrsiflora*	Leaves	1D NMR; ESIMS	Li et al. (2008)
5	Isoquercetrin	*E*. *thyrsiflora*	Leaves	1D NMR; ESIMS	Li et al. (2009)
6	Hyperoside	*E*. *thyrsiflora*	Leaves	1D NMR; ESIMS	Li et al. (2009)
7	Trifolin	*E*. *thyrsiflora*	Leaves	1D NMR; ESIMS	Li et al. (2009)
8	Astragalin	*E*. *thyrsiflora* *E. asperula*	Leaves Leaves	1D NMR; ESIMS HPLC	Li et al. (2009) Le et al. (2021)
9	Kaempferol 3-*O*-arabinosylgalactoside	*E*. *thyrsiflora*	Leaves	1D NMR; ESIMS	Li et al. (2009)
10	Quercetin 3-*O*-arabinosylgalactoside	*E*. *thyrsiflora*	Leaves	1D NMR; ESIMS	Li et al. (2009)
11	Kaempferol 3-rutinoside	*E. asperula*	Leaves	HPLC	Le et al. (2021)
12	Apigenin	*E. ovalifolia*	Leaves	1D NMR; ESIMS	Khattab et al. (2001)
13	Luteolin	*E. ovalifolia*	Leaves	1D NMR; ESIMS	Khattab et al. (2001)
14	Naringin	*E. macrophylla*	Fruit	HPLC	He (2018)
15	Ovalifolin	*E. ovalifolia*	Leaves	1D, 2D NMR; HRESIMS	Khattab et al. (2001)
16	(+)-Catechin hydrate	*E. macrophylla*	Fruit	HPLC	He (2018)
Phenylpropanoids					
17	Cinnamic acid	*E*. *thyrsiflora*	Leaves	1D NMR; ESIMS	Li et al. (2009)
18	Caffeic acid	*E*. *thyrsiflora* *E. asperula* *E. macrophylla*	Leaves Leaves Fruit	1D NMR; ESIMS HPLC 1D NMR, HRLCMS	Li et al. (2009) Le et al. (2021) Lu, (2016)
19	Ferulic acid	*E*. *thyrsiflora*	Leaves	1D NMR; ESIMS	Li et al. (2009)
20	*α*-Hydroxydihydrocaffeic acid	*E*. *thyrsiflora*	Leaves	1D NMR; ESIMS	Li et al. (2009)
21	Caffeic acid methyl ester	*E. macrophylla*	Fruit	1D NMR, HRLCMS	Lu (2016)
22	Ethyl caffeate	*E*. *thyrsiflora*	Leaves	1D NMR; ESIMS	Li et al. (2010b)
23	Ehretiate	*E. longiflora*	Root	1D, 2D NMR; HRESIMS	Chien et al. (2012)
24	*trans*-4-Hydroxycyclohexyl-2-*O*-*p*-coumaroyl *β*-D-glucopyranoside	*E. obtusifolia*	Whole plant	1D, 2D NMR; HRFABMS	Iqbal et al. (2005)
25	Methyl 2-*O*-feruloyl-1*α*-*O*-vanillactate	*E. obtusifolia*	Whole plant	1D NMR; HRFABMS	Iqbal et al. (2005)
26	Methyl rosmarinate	*E*. *thyrsiflora* *E. asperula* *E. macrophylla* *E. obtusifolia*	Leaves Leaves Fruit Whole plant	1D NMR; HRTOFMS HPLC 1D NMR, HRLCMS 1D NMR; HRFABMS	Li et al. (2008) Le et al. (2021) Lu, (2016) Iqbal et al. (2005)
27	Rosmarinic acid	*E*. *thyrsiflora* *E. asperula* *E. macrophylla* *E. obtusifolia*	Leaves Leaves Fruit Whole plant	1D NMR; ESIMS HPLC 1D NMR, HRLCMS 1D NMR; HRFABMS	Li et al. (2009) Le et al. (2021) Lu (2016) Iqbal et al. (2005)
28	Clinopodic acid B	*E. asperula*	Leaves	HPLC	Le et al. (2021)
29	Caffeic anhydride	*E. obtusifolia*	Whole plant	1D, 2D NMR; HRFABMS	Iqbal et al. (2005)
30	Icariside E_5_	*E*. *thyrsiflora* *E. ovalifolia*	Leaves Bark	1D NMR; ESIMS 1D NMR; FABMS	Li et al. (2009) Yoshikawa et al. (1995)
31	Ehletianol D	*E. ovalifolia*	Bark	1D, 2D NMR; FABMS	Yoshikawa et al. (1995)
32	1-(4-Hydroxy-3-methoxyphenyl)-2-{2-methoxy-4-[1-(*E*) propen-3-ol]-phenoxy}-propane-3-diol (*erythro*)	*E. ovalifolia*	Bark	1D NMR; FABMS	Yoshikawa et al. (1995)
33	1-(4-Hydroxy-3-methoxyphenyl)-2-{2-methoxy-4-[1-(*E*) propen-3-ol]-phenoxy}-propane-l,3-diol (*threo*)	*E. ovalifolia*	Bark	1D NMR; FABMS	Yoshikawa et al. (1995)
34	Ehletianol C	*E. ovalifolia*	Bark	1D, 2D NMR; FABMS	Yoshikawa et al. (1995)
35	Buddlenol B	*E. ovalifolia*	Bark	1D NMR; FABMS	Yoshikawa et al. (1995)
36	Ethyl lithospermate	*E. asperula*	Leaves	HPLC	Le et al. (2021)
37	Lithospermic acid B	*E*. *thyrsiflora* *E. asperula*	Leaves Leaves	1D NMR; ESIMS HPLC	Li et al. (2009) Le et al. (2021)
Simple phenolics					
38	Ehletianol A	*E. ovalifolia*	Bark	1D, 2D NMR; FABMS	Yoshikawa et al. (1995)
39	Ehletianol B	*E. ovalifolia*	Bark	1D, 2D NMR; FABMS	Yoshikawa et al. (1995)
40	2-Methoxyl benzoic acid octyl ester	*E*. *thyrsiflora*	Leaves	1D NMR; ESIMS	Li et al. (2010b)
41	*p*-Hydroxy benzoic acid	*E*. *thyrsiflora*	Leaves	1D NMR; ESIMS	Li et al. (2008)
42	Protocatechuic acid	*E. macrophylla*	Fruit	1D NMR, HRLCMS	Lu (2016)
43	Ehreticoumarinl	*E. longiflora*	Root	1D, 2D NMR; HRESIMS	Chien et al. (2012)
44	Ehretilactone A	*E. longiflora*	Root	1D, 2D NMR; HRESIMS	Chien et al. (2012)
45	Ehretiamide	*E. longiflora*	Root	1D, 2D NMR; HRESIMS	Chien et al. (2012)
46	Ehretioside B	*E. philippinensis*	Bark	1D, 2D NMR; HRFABMS	Simpol et al. (1994)
47	Prenylhydroquinone	*E. longiflora*	Root	1D NMR; ESIMS	Chien et al. (2012)
48	4-Hydroxy-3-prenylbenzoic acid	*E. longiflora*	Root	1D NMR; ESIMS	Chien et al. (2012)
49	Proglobeflowery acid	*E. longiflora*	Root	1D NMR; ESIMS	Chien et al. (2012)
50	Syringaldehyde	*E. longiflora*	Root	1D NMR; ESIMS	Chien et al. (2012)
Benzoquinones					
51	Microphyllone	*E. microphylla*	Aerial parts	1D, 2D NMR; ESIMS, X-ray	Agarwal et al. (1980)
52	Dehydromicrophyllone	*E. microphylla*	Aerial parts	1D, 2D NMR; HRFABMS	Yamamura et al. (1995)
53	Hydroxymicrophyllone	*E. microphylla*	Aerial parts	1D, 2D NMR; HRFABMS	Yamamura et al. (1995)
54	Cyclomicrophyllon	*E. microphylla*	Aerial parts	1D, 2D NMR; HRFABMS	Yamamura et al. (1995)
55	Allomicrophyllo	*E. microphylla*	Aerial parts	1D, 2D NMR; HRFABMS	Yamamura et al. (1995)
56	Ehretianone	*E. buxifolia*	Root Bark	1D, 2D NMR; ESIMS; X-ray	Selvanayagam et al. (1996)
57	Ehretiquinone	*E. longiflora*	Root	1D NMR; HRESIMS; X-ray	Chien et al. (2012)
Triterpenoids and sterols					
58	Ursolic acid	*E. microphylla*	Aerial parts	1D NMR; ESIMS	Yamamura et al. (1995)
59	*α*-Amyrin	*E. Asperula* *E*. *laevis* *E. cymosa* *E*. *rigida*	Leaves Bark Leaves Bark	HPLC 1D NMR; FABMS 1D NMR 1D NMR, HREIMS	Le et al. (2021) Dan and Dan (1982) Chaluma et al. (2018) Steyn (1998)
60	Bauerenol	*E*. *laevis*	Bark	1D NMR; FABMS	Dan and Dan (1982)
61	Bauerenyl acetate	*E*. *laevis*	Bark	1D NMR; FABMS	Dan and Dan (1982)
62	Ehretiolide	*E. longiflora*	Root	1D, 2D NMR; HRESIMS	Chien et al. (2012)
63	Oleanolic acid	*E. longiflora*	Root	1D NMR; ESIMS	Chien et al. (2012)
64	*O*-Acetyloleanolic acid	*E. longiflora*	Root	1D NMR; ESIMS	Chien et al. (2012)
65	*β*-Amyrin	*E. Asperula* *E. cymosa* *E. rigida*	Leaves Leaves Bark	HPLC 1D NMR 1D NMR, HREIMS	Le et al. (2021) Chaluma et al. (2018) Steyn (1998)
66	Lupeol	*E*. *laevis*	Bark	1D NMR; FABMS	Dan and Dan (1982)
67	Betulinic acid	*E*. *laevis*	Bark	1D NMR; FABMS	Dan and Dan (1982)
68	Betulin	*E*. *laevis*	Bark	1D NMR; FABMS	Dan and Dan (1982)
69	Stigmasterol	*E. buxifolia*	Root Bark	GC-MS	Selvanayagam et al. (1996)
70	Campesterol	*E. buxifolia*	Root Bark	GC-MS	Selvanayagam et al. (1996)
71	Stigmastanol	*E. buxifolia*	Root Bark	GC-MS	Selvanayagam et al. (1996)
72	*α*-Spinasterol	*E. buxifolia*	Root Bark	GC-MS	Selvanayagam et al. (1996)
73	Cholesterol	*E. buxifolia*	Root Bark	GC-MS	Selvanayagam et al. (1996)
74	*β*-Sitosterol	*E*. *thyrsiflora* *E. buxifolia*	Leaves Root Bark	TLC GC-MS	Li et al. (2010b) Selvanayagam et al. (1996)
75	Daucoster	*E*. *thyrsiflora*	Leaves	TLC	Li et al. (2010b)
Fatty acids					
76	Tetradecenoic acid, 2,3-dihydroxypropyl ester	*E*. *thyrsiflora*	Leaves	1D NMR; ESIMS	Li et al. (2010b)
77	(10*E*,12*Z*,15*Z*)-9-Hydroxy-10,12,15-octadecatrienoic acid methyl ester	*E. dicksonii*	Leaves	1D, 2D NMR; HREIMS	Dong et al. (2000)
78	(9*E*,11*E*)-13-0xo-9,11-tridecadienoic acid	*E. dicksonii*	Leaves	1D, 2D NMR; HREIMS	Dong et al. (2000)
79	Butyric acid	*E*. *laevis*	Leaves	TLC	Torane et al. (2009)
80	(9Z,11*E*)-13-0xo-9,11-tridecadienoic acid	*E. dicksonii*	Leaves	1D NMR; HREIMS	Dong et al. (2000)
81	(9*Z*,12*Z*,14*E*)-16-Hydroxy-9, 12, 14-octadecatrienoic acid	*E. dicksonii*	Leaves	1D, 2D NMR; HREIMS	Dong et al. (2000)
82	(9*Z*,12*Z*)-2,3-Dihydroxypropyl octadeca-9,12-dienoate	*E. longiflora*	Root	1D NMR; ESIMS	Chien et al. (2012)
83	(9*Z*,12*Z*,15*Z*)-2,3-Dihydroxypropyl octadeca-9,12,15-trienoate	*E. longiflora*	Root	1D NMR; ESIMS	Chien et al. (2012)
84	(9*Z*,11*E*)-13-0xo-9,11-ocatadecadienoic acid	*E. dicksonii*	Leaves	1D NMR; HREIMS	Dong et al. (2000)
85	(+)-(2*E*,6*E*)-Methyl-10,11-dihydroxy-3,7,11-trimethyl-2,6-dodecadienoate	*E. longiflora*	Root	1D NMR; ESIMS	Chien et al. (2012)
86	(9*Z*,11*E*)-13-Hydroxy-9,11-octadecadienoic acid	*E. dicksonii*	Leaves	1D NMR; HREIMS	Dong et al. (2000)
87	Methyl linoleate	*E. longiflora*	Root	1D NMR; ESIMS	Chien et al. (2012)
88	2,3-Dihydroxypropyl palmitate	*E. longiflora*	Root	1D NMR; ESIMS	Chien et al. (2012)
89	3-Methylbut-2-enoic acid	*E. longiflora*	Root	1D NMR; ESIMS	Chien et al. (2012)
90	1-Triacontanol	*E. rigida*	Bark	1D NMR, HREIMS	Steyn (1998)
91	Decanoic acid	*E*. *laevis*	Leaves	GC-MS	Velappan and Thangaraj (2014)
92	Phytol	*E*. *laevis*	Leaves	GC-MS	Velappan and Thangaraj (2014)
Others					
93	Allantoin	*E*. *thyrsiflora* *E. microphylla* *E. rigida*	Leaves Aerial parts Bark	1D NMR; ESIMS 1D NMR; ESIMS 1D NMR, HREIMS	Li et al. (2010b) Agarwal et al. (1980) Steyn (1998)
94	7-*O*-(*p*-methoxybenzoyl)-retronecanol	*E. aspera*	Leaves	1D NMR; ESIMS	Suri et al. (1980)
95	Ehretine	*E. longiflora*	Root	1D, 2D NMR; HRESIMS	Chien et al. (2012)
96	Simmondsin	*E. philippinensis*	Bark	1D, 2D NMR; HRFABMS	Simpol et al. (1994)
97	Ehretioside A1	*E. philippinensis*	Bark	1D, 2D NMR; HRFABMS	Simpol et al. (1994)
98	Ehretioside A2	*E. philippinensis*	Bark	1D, 2D NMR; HRFABMS	Simpol et al. (1994)
99	Ehretioside A3	*E. philippinensis*	Bark	1D, 2D NMR; HRFABMS	Simpol et al. (1994)
100	Phthalic acid	*E*. *laevis*	Leaves	GC-MS	Velappan and Thangaraj (2014)
101	Ehretilactone B	*E. longiflora*	Root	1D, 2D NMR; HRESIMS	Chien et al. (2012)

Note: NMR, nuclear magnetic resonance; HRESIMS, high resolution electrospray ionization mass spectroscopy; ESIMS, electron ionization mass spectrometry; FABMS, Fast-atom-bombardment Mass Spectrometry; GC-MS, Gas chromatography-mass spectrometry; TLC, Thin-layer chromatography; X-ray, X-ray crystallography.

### 3.1 Flavonoids

Flavonoids and their derivatives comprise the primary compounds of the *Ehretia* species. More than 16 compounds, including flavonoids and its glucoside (**1**–**13**, **15**), a flavanonol (**14**) and a flavanol (**16**) have been identified from leaves of *E. thyrsiflora*, *E*. *Asperula*, *E. ovalifolia* and fruits of *E. macrophylla*. Flavonoid glycosides comprise a diverse range of sugars, including glucose (Glc), galactose (Gla), arabinose (Ara) and rhamnose (Rha). Compounds **9**–**11** amd **14** have two different sugars. Ovalifolin (**15**) is a new isopentenyl flavonoid got from *E. ovalifolia* leaves (Khattab et al., 2001). (+)-Catechin hydrate (**16**) is a flavanol identified from the fruit of *E. macrophylla* (He, 2018). Each of them contains only hydroxyl groups and lacks methoxyl groups on their aromatic rings.

### 3.2 Phenypropanoids

This genus is rich in phenypropanoids (**17**–**37**), which usually show C_6_-C_3_ carbon skeleton. The compounds can be categorized into phenylpropionic acids and lignans and got from the fruit, leaves and bark of *Ehretia* species. Compounds **17**–**22** are the simple phenylpropionic acids. Ehretiate (**23**) is a new *trans*-icosanyl sinapate that has been isolated from the root of *E. longiflora* (Chien et al., 2012). Rosmarinic acid (**27**) is an ester of caffeic acid and 3,4-dihydroxyphenyllactic acid that possesses a diverse range of activities (Ijaz et al., 2023; Petersen, 2013; Petersen and Simmonds, 2003). The Labiatae and Boraginaceae family contain the greatest concentration of rosmarinic acid (Dresler et al., 2017). Three novel compounds were obtained from the dried whole plant of *E. obtusifolia*: *trans*-4-hydroxycyclohexyl-2-*O*-*p*-coumaroyl *β*-D-glucopyranoside (**24**), methyl 2-*O*-feruloyl-1*α*-*O*-vanillactate (**25**) and caffeic anhydride (**29**); Ehletianol C (**34**), buddlenol B (**35**) and ethyl lithospermate (**36**) are lignans containing 3 phenylpropanes (Le et al., 2021; Yoshikawa et al., 1995). Four phenylpropanes connected by C-C and C-O-C bonds combine to generate the lignan known as lithospermic acid B (**37**) (Li et al., 2009).

### 3.3 Simple phenolics

The novel phenolic compounds, called ehletianols A and B (**38** and **39**), were separated from the bark of *E. ovalifolia* and contain two sugars (Yoshikawa et al., 1995). Simple phenolics with carboxyl or ester groups are compounds **40**–**44**. The root of *E. longiflora* yielded ehretiamide (**45**), a novel phenolic containing acetyl group (Chien et al., 2012). Ehretioside B (**46**) is a new phenolic which connected with a cyanomethylene acid group (Simpol et al., 1994). Simple phenolics containing an isopentene or aldehyde group include prenylhydroquinone (**47**), 4-hydroxy-3-prenylbenzoic (**48**), proglobeflowery acid (**49**) (Chien et al., 2012).

### 3.4 Benzoquinones

Seven benzoquinones dimeric prenylbenzoquinone moieties were identified in the aerial portions of *E. microphylla*, the root bark of *E. buxifolia*, and the root of *E. longiflora*. The absolute configuration of three novel compounds-microphyllone (**51**), ehretianone (**56**) and ehretiquinone (**57**)-was determined using X-ray crystallographic analysis (Agarwal et al., 1980; Chien et al., 2012; Selvanayagam et al., 1996). Compounds **52**–**55** are the congeners of microphyllone (**51**) (Yamamura et al., 1995).

### 3.5 Triterpenoids and sterols

To now, only 3 types of triterpenoids (ursulane type, oleanane type and lupinane type) were reported from the *Ehretia* genus. These are all common triterpenoids, except for ehretiolide (**62**) (Chien et al., 2012). Ehretiolide (**62**) is a new ursulane type triterpenoid containing the O-(*E*)-4′-methoxylcinnamoyl and lactone groups. Additionally, the GC-MS detected every sterol with the exception of the daucoster (**75**), which was determined by TLC (Li et al., 2010b).

### 3.6 Fatty acids

This genus has been observed to contain 17 fatty acids. Fatty acid esters make up the majority of these molecules, with fatty alcohols making up the minority.

### 3.7 Others

A derivative of hydantolurea, allantoin (**93**) was isolated from *E. thyrsiflora* leaves, *E. microphylla* aerial parts, and *E. rigida* bark (Agarwal et al., 1980; Li et al., 2010b; Steyn, 1998). A novel pyrrolizidine, 7-*O*-(*p*-methoxybenzoyl)-retronecanol (**94**) has been isolated from *E. aspera* leaves (Suri et al., 1980). Ehretine (**95**) is a new alkaloid which having a cyclohex-2-en-1-one moiety (Chien et al., 2012). Simmondsin (**96**), ehretioside A1 (**97**), ehretioside A2 (**98**), ehretioside A3 (**99**) are four cyanoglucosides which were isolated from *E. philippinensis* bark (Simpol et al., 1994).

## 4 Biological activity

### 4.1 Antioxidant activity

The ethyl acetate (EA) extracts from the bark of *E. acuminata* exhibited potent antioxidant activity, with IC_50_ values of 22 μg/mL, 26 μg/mL, and 140 μg/mL for ABTS, DPPH, and NO radical scavenging assays, respectively (Table 4). In comparison, the standard ascorbic acid (AA) had IC_50_ values of 28 μg/mL, 30 μg/mL, and 175 μg/mL, respectively. This suggests that the bark of this plant possesses significant health benefits due to its antioxidant properties, as supported by previous research (Kaur et al., 2019). The EA extract from the fruit of *E. acuminata* also demonstrated strong free radical scavenging capacity, as reported by Shukla group, with IC_50_ values of 40 μg/mL, 50 μg/mL, and 380 μg/mL for ABTS, DPPH, and NO assays, respectively. For the same assays, the standard AA had IC_50_ values of 23 μg/mL, 27 μg/mL, and 230 μg/mL, respectively (Shukla et al., 2019b). Notably, the EA extract from the leaves of *E. acuminata* exhibited the highest antioxidant activity, with IC_50_ values of 90 μg/mL, 99 μg/mL, and 250 μg/mL for ABTS, DPPH, and NO assays, respectively (Shukla et al., 2019a). *In vitro* antioxidant and radical scavenging assays conducted on *E. serrata* fruits and leaves revealed that the EA fraction of the leaf extract exhibited the highest activity. This is attributed to its high phenolic content. Additionally, the EA fraction of the fruit extract showed the highest lipid peroxidation value, followed by the leaf fraction. Significant free radical scavenging potential was also observed in the chloroform (CH) and 1-butanolic fractions of the leaf extract (Zara et al., 2012). R28 cells were used to evaluate the protective effects of phytochemicals and ethanolic extracts from *E. asperula* leaves against oxidative stress-induced retinal cell loss and excitotoxicity. The results indicated that both 70% and 95% ethanolic leaf extracts enhanced cell viability under these conditions. Furthermore, methyl rosmarinic acid (**26**) and rosmarinic acid (**27**) were particularly effective in preventing retinal cell death and increasing ROS levels in cells exposed to glutamate/BSO-induced excitotoxicity/oxidative stress. These findings suggest that *E. asperula* leaves may have therapeutic potential for treating retinal degeneration (Le et al., 2021).

**TABLE 4:** Bioactivities attributed to the species of *Ehretia* genus.

Bioactivities	Species	Parts used	Extract and/or compounds	Results	Reference
Antioxidant	*E. acuminata*	Bark	EA extract	IC_50_ values of 22, 26, and 140 μg/mL for ABTS, DPPH, and NO radical scavenging activity *in vitro*	Kaur et al. (2019)
		Leaves	EA extract	IC_50_ values of 90, 99, and 250 μg/mL for ABTS, DPPH, and NO radical scavenging activity *in vitro*	Shukla et al. (2019a)
		Fruit	EA extract	IC_50_ values for the ABTS, DPPH, and NO assays were 40, 50, and 380 μg/mL *in vitro*	Shukla et al. (2019b)
	*E. serrata*	Leaves	EA extract	ABTS (TEAC 1.76 ± 0.004 µmol), DPPH (EC_50_ 120.499 μg/mL), FRAP (270.44 ± 1.00 µmol of ascorbic acid equivalent, AAE), Phosphomolybdate (156.92 ± 4.63 μg/mL of AAE) *in vitro*	Zara et al. (2012)
	*E. asperula*	Leaves	Methyl rosmarinic acid (**26**)	Under the condition of glutamate/BSO-induced cytotoxicity, **26** (100 μM) significantly improved the survival rate of R28 cells	Le et al. (2021)
			Rosmarinic acid (**27**)	Under the condition of glutamate/BSO-induced cytotoxicity, **27** (33 μM) significantly improved the survival rate of R28 cells	
	*E. macrophylla*	Peels	Frozen acetone extract	PSC and ORAC values of 859.85 ± 70.32 μmol vit. C equiv./g, DW, 2877.01 ± 163.80 μmol trolox equiv./g *in vitro*	Lu (2016)
		Fruit	Polyphenols	Showed significant free radical scavenging ability: DPPH (EC_50_ = 0.32 ± 0.03 mg/mL), and TEAC (4,134 ± 9.7 μM TE/g dry extract)	Deng et al. (2020)
			Methyl rosmarinate (**26**)	ORAC, PSC, and CAA values (wash) were 28,976.58 ± 3,589.36 μmol TE/g DW, 32,139.75 ± 3,979.76 μmol EVc/g DW, and 317.77 ± 36.69 μmol QE/100μmol, respectively	He (2018)
	*E. serrata*	Stem bark	Methanolic extract	Exhibited significant (94.1%) DPPH inhibition at dose level 1,000 μg/mL after 60 min	Huma (2019)
		Fruit	Methanolic extract	Exhibited significant (85.01%) DPPH inhibition at dose level 1,000 μg/mL after 60 min	
	*E. tinifolia*	Fruits	Methanolic extract	DPPH, 303.8 mg EVC/100 g f.w.; ABTS, 84.1 mg EVC/100 g f.w. (EVC means equivalents of vitamin C) *in vitro*	Pío-León et al. (2012b)
	*E. laevis*	Flowers	Hydroalocoholic (70%) extract	IC_50_ values of 56.5 and 478.8 μg/mL for DPPH, and NO radical scavenging activity *in vitro*	Rangnathrao and Shanmugasundaram (2019)
	*E. microphylla*	Aerial parts	CH and EA extracts	Significantly decreased CAT, SOD, GSH and GPx levels *in vivo*	Yuvaraja et al. (2021)
	*E. cymosa*	Leaves	Methnanol extract	IC_50_ values of 0.47, 0.49, and 0.55 mg/mL for DPPH, ABTS and -OH radical scavenging activity *in vitro*	Ogundajo and Ashafa (2017)
			EA extract	IC_50_ values of 0.61 and 1.68 mg/mL for metal chelating and superoxide anion scavenging activity *in vitro*	
		Whole plant	70% Ethanolic extract	IC_50_ values of 0.489 μg/mL for DPPH radical scavenging activity *in vitro* (BHT and AA was 0.403 and 0.032 μg/mL, respectively)	Sarkodie et al. (2015)
	*E. longiflora*	Root	Ehretiquinone (**57**)	Inhibitory effects on *N*-formyl methionyl leucyl phenylalanine (fMLP)-induced superoxide production (IC_50_ = 0.36 ± 0.03 μM)	Chien et al. (2012)
Anti-diabetic	*E. acuminata*	Bark	CH extract	IC_50_ values of 260 and 265 μg/mL for α-amylase and α-glycosidase inhibitory activity, respectively (Acarbose with IC_50_ values of 40.25 and 38.45 μg/mL, respectively)	Kaur et al. (2022)
		Leaves	CH extract	IC_50_ values of 43.35 and 42.90 μg/mL for α-amylase and α-glycosidase inhibitory activity, respectively	Shukla et al. (2021)
	*E. anacua*	Leaf	Aqueous extract	Treating alloxan-induced rats with 50, 100 and 200 mg/kg bw extract caused a significant decrease in the blood glucose level	Abimbola et al. (2021)
	*E. cymosa*	leaves	Methanol and EA extracts	Against α-amylase (2.11 and 2.75 mg/mL) and α-glucosidase (0.66 and 0.60 mg/mL), respectively	Ogundajo and Ashafa, (2017)
		Whole plant	70% ethanolic extract	Different doses of the extract administered (30 mg/kg, 60 mg/kg and 90 mg/kg) gave statistically significant reduction in FBGL of the rats (streptozotocin induced)	Sarkodie et al. (2015)
	*E. macrophylla*	Fruit	Polyphenols	Suppressing the activities of α-glucosidase and α-amylase, increasing glucose consumption, glycogen accumulation, and GYS2, and reducing the effects of G6Pase and PEPCK	Deng et al. (2020)
	*E. tinifolia*	Fruit	Polyphenols	Against α-amylase (0.17 mg/mL) and α-glucosidase (5 mg/mL), respectively	Monroy-García et al. (2021)
Analgesic and anti-inflammatory	*E. laevis*	Leaves	Methanol extract	The herbal hydrogel HEL3 exhibited an impressive 78.75% and 79.51% inhibition, respectively, compared to the standard (indomethacin), which exhibited an inhibition of 80.24% and 81.12% at 360 min in carrageenan induced rat paw edema method and formalin induced rat paw edema method	Memon et al. (2022)
	*E. tinifolia*	Whole plant	Methanol extract	Inhibited the LPS-induced production of pro-inflammatory cytokines, including TNF-α, IL-1β, and IL-6	Lim et al. (2023)
	*E. acuminata*	Leaves	PE and EA extracts	Highest potential was shown by EA extract (IC_50_ 290 μg/mL) and lowest in PE extract (IC_50_ 750 μg/mL) using spectrophotometric method	Shukla et al. (2021)
		Bark	EA and ethanol extracts	Showed significant anti-inflammatory effects (evaluated with the egg albumin of hen *in vitro*) with IC_50_ values of 170 and 172 μg/mL, respectively	Kaur et al. (2022)
	*E. dicksonii*	Fresh leaves and twigs	(10E, 12Z, 15Z)-9-hydroxy-10, 12, 15-octadecatrienoic acid methyl ester (**77**)	Demonstrated a 43% inhibitory effect on TPA-exposed inflammation in the ears of mouse when administered at a dose of 500 μg	Dong et al. (2000)
			Compounds **78**, **80**, **81**, **86** and **84**	Exhibited potent activity with, IE_500 μg_ of 32%, 19%, 39%, 63%, and 79%, respectively	
	*E. cymosa*	Leaves	80% methanol extract	80% methanol extract, aqueous, ethyl acetate and chloroform fractions of *E. cymosa* demonstrated significant analgesic and anti-inflammatory activities, evaluated using acetic acid-induced writhing and hot plate tests, carrageenan-induced paw edema and cotton-pellet-induced granuloma models, respectively	Ashagrie et al. (2023)
	*E. macrophylla*	Fruit	EA and CH extracts	The EA and CH fractions showed potent anti-inflammatory activity (LPS stimulation of RAW264.7 cells model), with an IC_50_ value of 19.10 ± 0.31 μg/mL and 19.48 ± 0.25 μg/mL for inhibiting NO release	He (2018)
			Methyl caffeate and methyl rosmarinate	Two compounds notably reduced the levels of TNF-α, IL-1β, and iNOS at 2 μg/mL and 1.25 μg/mL, respectively	
	*E. laevis*	Leaves	CH, methanolic, and aqueous extracts	These extracts showed significant anti-inflammatory activity by reducing paw volume at different doses	Jyothirmai et al. (2016)
	*E. obtusifolia*	Stem bark	Methanolic extract	Highly significant analgesic effect at all dose level 100 mg/kg, 200 mg/kg and 300 mg/kg over 60 min	Huma (2019)
Hepatoprotective	*E. laevis*	Flowers	70% hydroalcoholic and EA extract	Demonstrated substantial dose-dependent protection against alterations in serum ASAT, ALAT, ALP, and TP at oral doses of 100 and 200 mg/kg	Rangnathrao and Shanmugasundaram (2019)
	*E. microphylla*	Aerial parts	CH and EA extracts	CH and EA extracts significantly shielded rats from liver toxicities caused by paracetamol. SGOT, SGPT, and ALP levels, as well as triglyceride and total cholesterol levels, were elevated in rats with liver injury caused by paracetamol	Yuvaraja et al. (2021)
Muscle relaxant and antispasmodic	*E. acuminata*	Bark	Water, ethanol, and chloroform extracts	These extracts also showed antispasmodic (11 ± 1, 9 ± 1, and 11 ± 1), analgesic (10 ± 1, 16 ± 1, and 11 ± 1), and muscle relaxant (6 ± 1, 5 ± 1, and 5 ± 1) potential at 300 mg/kg	Jan et al. (2023)
	*E. obtusifolia*	Stem bark	Methanolic extract	Exhibited highly significant results at dose of 200 and 300 mg/kg in swiss albino mice	Huma (2019)
Antibacterial	*E. serrata*	Leaves	Methanolic extract	Exhibited antimicrobial activity against all the tested microorganisms including *Azospirillum lipoferum*, *Escherichia coli*, *Pseudomonas aeruginosa*, *Stenotrophomonas maltophilia* and *Enterococcus* sp. with ZOI ranged from 10.3 to 29.0 mm. Additionally, both the methanolic extract and its sub-fractions exhibited MIC values ranging from 0.8 to 5.1 mg/mL against the tested bacteria	Waheed et al. (2019)
				Exhibited a substantial antibacterial zone (7 mm) at a dosage level of 1,000 μg/mL, in contrast to the reference drug’s (31.5 mm)	Huma (2019)
	*E. longiflora*	Root	Prenylhydroquinone (**47**) and ehretiquinone (**57**)	Showed antitubercular activity against *Mycobacterium tuberculosis* strain H37Rv with MIC values of 25.0 and 26.2 μg/mL, respectively	Chien et al. (2012)
	*E. acuminata*	Leaf	Ethanol extract	Exhibited the most extensive zone of inhibition (12–18 mm) against a variety of food poisoning bacteria (*L. monocytogenes*, *S. aureus*, *E. coli* and *P. aeruginosa*)	Shukla et al. (2021)
	*E. cymosa*	Whole plant	70% Ethanolic extract	Showed inhibitory activity against *P. aeruginosa*, *E. coli*, *B. subtilis* and *S. aureus*	Sarkodie et al. (2015)
Anticancer	*E. laevis*	Leaves	Aqueous extract	The nanoparticles (synthetic silver nanoparticles, which had a spherical form and a diameter of 25–35 nm) exhibited a median lethal concentration of 12.7 μg/mL and 14.5 μg/mL against HeLa, human cervical cancer cells, and MCF-7 human breast cancer cells, respectively	Panja et al. (2020)
	*E. macrophylla*	Fruit	EA and CH extracts	Exhibited significant anti-proliferation activity in tested dosage	He, (2018)
			Methyl caffeate (**21**)	Demonstrated stronger anti-proliferation effect on Caco-2 cells (EC_50_ = 132.73 ± 18.19 μM) and HepG2 cells (EC_50_ = 48.35 ± 0.61 μM)	
	*E. microphylla*	Leaves with flower buds	Ethanolic extract	Exhibited a significant and highly selective antiproliferative effect on HT-29 (IC_50_ value 130.89 ± 13.99 μg/mL) and Caco-2 (IC_50_ value 52.49 ± 8.81 μg/mL) cells	Kudera et al. (2021)
		Leaves	CH extract	Displayed 85.55% and 77.93% inhibition against MCF-7 and A-549 cancer cells at 50 μg/mL, respectively	Sharma et al. (2022)
	*E. macrophylla*	Fruit	Polyphenols	Free and bound extracts powerfully inhibited the proliferation of HepG2 cells in a dose-dependent manner	Deng et al. (2020)
	*E. tinifolia*	Fruit	Polyphenols	IC_50_ values of 0.99, 1.36 and 0.82 mg/mL for MCF-7, HeLa, and HT-29 cells, respectively	Monroy-García et al. (2021)
Antihemolytic	*E. acuminata*	Leaves	EA extract	Demonstrated the most potential for antihemolytic activity, with an IC_50_ of 90 μg/mL	Shukla et al. (2021)
	*E. tinifolia*	Fruits	Polyphenols	Shown to possess the effect of hemolysis inhibition (IC_50_ = 58.55 ± 2.4 μg/mL)	Monroy-García et al. (2021)
Anti-arthritic	*E. laevis*	Leaves	Methanolic extract	In mice, it (500 mg/kg) inhibited the rise in paw volume by 56%, paw edema to 60%, helped maintain the body weight, and regulated changes in hematological parameters	Velappan and Thangaraj (2014)
Wound healing	*E. laevis*	Fresh leaves	Paste was prepared in mortar and pestle	With healing times ranging from 7 days to a maximum of 66 days, it shows considerable healing properties in both infected and non-infected, chronic and fresh wounds	Thakre et al. (2016)
		Leaves	CH extract	Had the same wound healing property as Povidon Iodine ointment in animal model	Harne et al. (2021)
	*C. retusa* (*E. microphylla*)	root, stem, and leaves	Alcoholic extract	Swiss albino mice were used for the evaluation of wound healing activity. The ointment prepared from different parts showed significant effectiveness in wound contraction and faster wound closure compared to the standard Nitrofurazone (0.2%) ointment	Mageswari et al. (2012)
Anti-amoebic	*E. laevis*	Leaves	Create the synthetic silver nanoparticles	Treated for 72 h, it killed 70% ± 10.24% of *Culex quinquefasciatus* larvae at a dosage of 25 μg/mL. Within 8 h, the nanoparticles reduced Congo red by approximately 85% at a concentration of 200 μg/mL	Panja et al. (2020)
Lipoxygenase inhibitory	*E. dicksonii*	Fresh leaves and twigs	Compounds **77**, **81**, **84** and **86**	Exhibited inhibitory activity against soybean lipoxygenase at a concentration of 10 μg/mL	Dong et al. (2000)
	*E. obtusifolia*	Whole plant	Compounds **24**–**27** and **29**	Compounds **24**–**27** and **29** demonstrated concentration-dependent inhibition of lipoxygenase, with *K*_i_ values ranging from 0.85 to 57.6 μM. Compounds **26** and **27** were the most potent, having *K*_i_ values of 0.85 μM and 1.0 μM, respectively	Iqbal et al. (2005)
Modulation of gut microbiota	*E. macrophylla*	Fruit	Polysaccharide (EWMFP)	The effects on human gut microbiota were studied using an *in vitro* fermentation model simulating human colon micro-ecosystem. In comparison to inulin, EWMFP can alter gut microbial compositions differently and yields higher concentration of butyrate by the end of fermentation	Deng et al. (2020)
Others	*E. buxifolia*	Root bark	Erhetianone (**56**)	The antisnake venom effect of **56** was determined by calculating the LD_50_ of *E. carinatus* venom in mice that was administered subcutaneously	Selvanayagam et al. (1996)
	*E. philippinensis*	Bark	Butanolic and EA extracts	Exhibited anti-histamine releasing activity	Simpol et al. (1994)
	*E. microphylla*	Whole plant	Powder	1,000 mg/kg of powdered increased the levels of FSH, LH, and estradiol significantly in female Wistar albino rats	Aarthi et al. (2012)

The high phenolic content of *E. macrophylla* peel extracts is a key factor contributing to their remarkable antioxidant activity, as evidenced by PSC and ORAC values of 859.85 ± 70.32 μmol vit. C equiv./g and 2877.01 ± 163.80 μmol trolox equiv./g, respectively (Lu, 2016). According to Huma, there was a notable reduction in DPPH after 60 min (94.1%) in the stem bark extract of *E. serrata* and 30 min (85.01%) in the fruit extract of *E. obtusifolia* at the dosage level of 1,000 μg/mL (Huma, 2019). Pío-León research group demonstrated that the antioxidant activity of *E. tinifolia* fruits was comparable to or higher than that of various tropical fruits, including guava, orange, and prickly pears. The 70% ethanolic extract of *E. laevis* flowers exhibited robust antioxidant effects, as shown by DPPH and NO reducing power assays (Rangnathrao and Shanmugasundaram, 2019). Compared to rodents subjected to paracetamol-induced toxicity, the CH and EA extracts of *E. microphylla* significantly increased antioxidant parameters (CAT, SOD, GSH, and GPx) in rats (Yuvaraja et al., 2021). The methanol fraction of *E. cymosa* leaves exhibited significantly stronger (P < 0.05) scavenging activities for DPPH (0.47 mg/mL), ABTS (0.49 mg/mL), and -OH radical (0.55 mg/mL) (Ogundajo and Ashafa, 2017). Two polyphenols found in *E. tinifolia* Linnaeus, TEAC (4,134 ± 9.7 μM TE/g dry extract) and DPPH (EC_50_ = 0.32 ± 0.03 mg/mL), demonstrated significant free radical scavenging capacity (Deng et al., 2020). After extracting active compounds from the dry fruit of *E. macrophylla* using 70% ethanol, four distinct solvents (EA, n-butanol, CH, and PE) were used to remove the remaining contents. Methyl rosmarinate (**26**) showed strong anti-oxidation action, with ORAC, PSC, and CAA values of 28,976.58 ± 3,589.36 μmol TE/g DW, 32,139.75 ± 3,979.76 μmol EVc/g DW, and 317.77 ± 36.69 μmol QE/100μmol, respectively. The EA fraction demonstrated strong antioxidant activity, with higher ORAC and PSC values compared to methyl caffeate (**21**) (He, 2018). Ehretiquinone (**57**), isolated from the root of *E. longiflora*, inhibited superoxide formation generated by N-formyl methionyl leucyl phenylalanine (fMLP) with an IC_50_ value of 0.36 ± 0.03 μM (Chien et al., 2012).

### 4.2 Anti-diabetic activity

The antidiabetic potential of various bark extracts of *E. acuminata* was evaluated *in vitro* using α-amylase and α-glycosidase inhibition assays. Among the tested extracts, only the CH extract demonstrated significant inhibitory activity against both enzymes, with IC_50_ values of 43.35 μg/mL for α-amylase and 42.90 μg/mL for α-glycosidase. These findings suggest that *E. acuminata* bark contains bioactive compounds with promising antidiabetic property (Kaur et al., 2022). In a separate study, researchers investigated the effects of an aqueous extract of *E. anacua* leaves on alloxan-induced diabetic rats. Histological examination of pancreatic tissues revealed that blood glucose levels were significantly higher in diabetic rats compared to the control group. Treatment with the aqueous extract at doses of 50, 100, and 200 mg/kg reduced blood glucose levels and preserved pancreatic histoarchitecture. Microscopic examination of pancreatic tissue showed significant architectural damage in the alloxan-treated group, while the plant extract-treated group exhibited normal architecture. This indicates that the extract mitigates alloxan-induced toxicity. These results support the use of the extract as a dietary supplement with hypoglycemic and antidiabetic activities for functional foods (Abimbola et al., 2021). The bioactive compounds in *E. cymosa* leaves were also studied for their antidiabetic properties. Methanol and EA fractions exhibited statistically significant inhibition (P < 0.05) against α-amylase (IC_50_: 2.11 mg/mL and 2.75 mg/mL, respectively) and α-glucosidase (IC_50_: 0.66 mg/mL and 0.60 mg/mL, respectively). Additionally, the methanol fraction inhibited α-amylase through a competitive mechanism and α-glucosidase through a noncompetitive mechanism. These findings suggest that *E. cymosa* leaves contain bioactive compounds with therapeutic potential for diabetes treatment (Ogundajo and Ashafa, 2017). Antihyperglycemic efficacy of a 70% ethanolic fraction of the whole *E. cymosa* plant was investigated *in vivo* using Sprague Dawley rats. Additional *ex vivo* experiments were performed to determine the modulatory effects on the absorption of glucose in the intestines. Irrespective of the dosage, the extract substantially decreased the fasting blood glucose level and equally reduced the amount of glucose absorbed by the rat intestinal sacs. The findings validate the conventional application of *E. cymosa* extract as a pharmaceutical treatment for diabetes in susceptible mice (Sarkodie et al., 2015). Phenolic compounds from *E. macrophylla* fruit exhibited effective hypoglycemic activity by inhibiting α-glucosidase and α-amylase, increasing glucose consumption and glycogen accumulation, and reducing G6Pase and PEPCK activity. Thus, E. macrophylla fruits have the potential to enhance human health as metabolites in functional foods, offering further health and economic benefits (Deng et al., 2020). *E. tinifolia* extracts showed no inhibitory effect on lipase but selectively inhibited α-glucosidase and α-amylase (Monroy-García et al., 2021). Sequential Soxhlet extraction of *E. acuminata* leaves yielded both nonpolar and polar extracts, which were evaluated for antidiabetic potential using spectrophotometric analysis. The CH extract had the highest antidiabetic activity, with an IC_50_ ranging from 260 to 265 μg/mL (Shukla et al., 2021).

### 4.3 Analgesic and anti-inflammatory activity

The volume of the paw edoema was substantially reduced in the carrageenan- and formalin-induced rat paw edoema methods when the paw edoema was generated by the herbal hydrogel containing the methanol extract of *E. laevis* leaves (HEL3). HEL3 exhibited 78.75% and 79.51% inhibition, respectively, compared to the standard indomethacin, which showed 80.24% and 81.12% inhibition at 360 min. HEL3 demonstrated potent anti-inflammatory and wound healing properties (Memon et al., 2022). The mechanisms underlying the anti-inflammatory properties of the methanol extract of *E. tinifolia* (ETME) were elucidated. ETME significantly increased total GSH levels and decreased pro-inflammatory cytokine and NO production. Additionally, ETME reduced the production of pro-inflammatory cytokines such as TNF-α, IL-1β, and IL-6 in response to LPS. The study’s results suggest that ETME may be capable of protecting Kupffer cells from NF-κB and MAPKs, as well as LPS-induced oxidative stress and heightened inflammatory responses can be mitigated through the activation of the antioxidant pathway involving Nrf2 and HO-1 (Lim et al., 2023). The spectrophotometric technique was employed to evaluate the anti-inflammatory properties of the leaf extracts (PE, CH, EA, EOL, and aqua) of *E. acuminata*. Shukla group reported that the EA extract exhibited the most potent anti-inflammatory activity (Shukla et al., 2021). When tested *in vitro* with hen egg albumin, the EA and ethanol extracts of *E. acuminata* bark shown potent anti-inflammatory properties (IC_50_ values of 170 μg/mL and 172 μg/mL, respectively). According to the findings, the bark of *E. acuminata* contains chemicals that show promise as probable anti-inflammatory agents (Kaur et al., 2022). Compound **77**, extracted from *E. dicksonii*, demonstrated a 43% inhibitory effect on TPA-exposed inflammation in the ears of mouse when administered at a dose of 500 μg. Compounds **78**, **80**, **81**, **86** and **84** exhibited potent activity with, IE_500 μg_ of 32%, 19%, 39%, 63%, and 79%, respectively (Dong et al., 2000). Acetic acid-induced writhing and hot plate experiments, carrageenan-induced paw edoema, and cotton-pellet-exposed granuloma models were employed to evaluate the analgesic and anti-inflammatory properties of *E. cymosa* leaves. The findings of this investigation substantiate the conventional application of *E. cymosa* in the management of a diverse array of inflammatory and painful conditions. Specifically, the 80% methanol, aqueous, EA, and CH extracts of the plant demonstrated substantial analgesic and anti-inflammatory effects (Ashagrie et al., 2023). The four distinct solvents (EA, *n*-butanol, CH, and PE) were used to extract the compounds that were shown to be effective from the dried fruit of *E. macrophylla* after 70% ethanol was used. Strong anti-inflammatory activity was demonstrated by the EA and CH fractions, with IC_50_ values for preventing NO release of 19.10 ± 0.31 μg/mL and 19.48 ± 0.25 μg/mL, respectively.

Methyl caffeate and methyl rosmarinate, two compounds that were extracted from *E. macrophylla*, showed better anti-inflammatory properties than the other four fractions; these compounds notably reduced the levels of TNF-α, IL-1β, and iNOS at 2 μg/mL and 1.25 μg/mL, respectively (He, 2018). Jyothirmai group demonstrated that the CH, methanolic, and aqueous extracts of *E. laevis* exhibited significant anti-inflammatory efficacy by reducing paw volume at varying concentrations (Jyothirmai et al., 2016). With the exception of stem bark, *E. serrata* demonstrated a highly significant analgesic effect at all dose levels (100, 200, and 300 mg/kg) over a 60-min period. The stem bark of *E. obtusifolia* exhibited highly significant (P < 0.01) results at all concentrations after 60 min. The anti-inflammatory effects of *E. serrata* and *E. obtusifolia* were found to be highly significant (P < 0.01) at all concentrations, including 100 mg/kg, 200 mg/kg, and 300 mg/kg, after two and 3 h (Huma, 2019).

### 4.4 Hepatoprotective activity

The hepatoprotective effect of the hydroalcoholic (70% ethanol) and EA fractions of *E. laevis* flowers were evaluated using Wistar rats. Both extracts demonstrated significant dose-dependent protection against alterations in serum aspartate ASAT, ALAT, ALP, and TP at oral doses of 100 and 200 mg/kg. Additionally, they provided dose-dependent protection against liver tissue modifications, such as necrosis, fatty degeneration, and lymphatic infiltration, induced by paracetamol. The EA fraction exhibited superior activity compared to the hydroalcoholic extract. These findings suggest that *E. laevis* extracts have promising potential as preventive treatments for liver damage (Rangnathrao and Shanmugasundaram, 2019). Similarly, the CH and EA extracts of *E. microphylla* significantly protected rats from paracetamol-induced liver toxicities. Elevated levels of SGOT, SGPT, ALP, triglycerides, and total cholesterol in paracetamol-induced liver injury were effectively reduced by pretreatment with *E. microphylla* extracts (CH and EA, 200 mg/kg), comparable to silymarin (100 mg/kg). In summary, *E. microphylla* extracts demonstrated substantial hepatoprotective benefits in the context of paracetamol-induced liver injury in rats. The hepatoprotective properties of *E. microphylla* may be attributed to the presence of flavonoids and phenolic compounds (Yuvaraja et al., 2021).

### 4.5 Muscle relaxant and antispasmodic activity

The bark extracts of *E. acuminata* exhibited concentration-dependent increases in intestinal motility in experimental animals. These extracts also demonstrated antispasmodic, analgesic, and muscle relaxant activities at 300 mg/kg, with no observed acute toxic effects in tested mice. Specifically, the antispasmodic activity was measured at 11 ± 1, 9 ± 1, and 11 ± 1; analgesic activity at 10 ± 1, 16 ± 1, and 11 ± 1; and muscle relaxant activity at 6 ± 1, 5 ± 1, and 5 ± 1. This study provides significant evidence supporting the pharmacological use of *E. acuminata* as an analgesic, antispasmodic, and muscle relaxant (Jan et al., 2023). Additionally, the methanolic extracts of the fruit, stem bark, and leaves of *E. serrata* and *E. obtusifolia* were evaluated for antispasmodic activity in Swiss albino rodents. The methanolic extract of the stem bark showed highly significant results (P < 0.01) at concentrations of 200 and 300 mg/kg. *E. obtusifolia* exhibited enhanced smooth muscle relaxation at low doses. The highly significant (P < 0.01) activities of the leaf (200 and 300 mg/kg), fruit (300 mg/kg), and stem bark extracts (all concentrations) scientifically validated their ethnopharmacological use as antispasmodic agents (Huma, 2019).

### 4.6 Antibacterial activity

The antibacterial potential of phytoconstituents was examined by assessing their ability to disrupt bacterial cell permeability and inhibit bacterial growth. The methanolic extract of *E. serrata* leaves demonstrated effective antibacterial activity against five tested bacteria, with ZOI ranging from 10.3 to 29.0 mm. The MIC values ranged from 0.8 to 5.1 mg/mL (Waheed et al., 2019). The CH, methanolic, and aqueous extracts of *E. laevis* exhibited exceptional antibacterial efficacy against both gram-positive and gram-negative bacteria, with the methanolic fraction showing the most potent activity (Jyothirmai et al., 2016). Two compounds, prenylhydroquinone (**47**) and ehretiquinone (**57**), were isolated from the methanolic fraction of *E. longiflora* root. These compounds exhibited antitubercular effect inhibit *Mycobacterium tuberculosis* strain H37Rv, with MIC values of 26.2 and 25.0 μg/mL, respectively (Chien et al., 2012). The ethanol extract of *E. acuminata* leaves exhibited the most extensive ZOI (12–18 mm) against various food poisoning bacteria (Shukla et al., 2021). The agar diffusion assay revealed that the 70% ethanolic fraction of the entire plant of *E. cymosa* inhibited *P. aeruginosa*, *E. coli*, *B. subtilis*, and *S. aureus* (Sarkodie et al., 2015). The extract exhibited inhibitory effect against *P. aeruginosa, E. coli, B. subtilis,* and *S. aureus* (Sarkodie et al., 2015). The methanolic extract of *E. serrata* leaves exhibited a substantial antibacterial zone (7 mm) at a dosage level of 1,000 μg/mL, in contrast to the reference drug’s (31.5 mm). The leaf extract of *E. obtusifolia* exhibited a more potent antibacterial zone (7 mm) than the reference medication (30.2 mm) at a dosage level of 1,000 μg/mL (Huma, 2019).

### 4.7 Anticancer activity

The leaves of *E. laevis* were utilized to synthesize spherical silver nanoparticles with diameters ranging from 25 to 35 nm. These nanoparticles exhibited potent anticancer activity, demonstrating high stability in solution and significant cytotoxicity. Specifically, the median lethal concentrations (LC_50_) for HeLa and MCF-7 cells were 12.7 μg/mL and 14.5 μg/mL, respectively (Panja et al., 2020). The effective compounds were extracted from *E*. *macrophylla* fruit by 70% ethanol, and then extracted with four different solvents. The EA and CH fractions exhibited significant anti-proliferation activity in tested dosage. Methyl caffeate (**21**) demonstrated stronger anti-proliferation effect on Caco-2 cells (EC_50_ = 132.73 ± 18.19 μM), while EC_50_ value of methyl caffeate on HepG2 cells (EC_50_ = 48.35 ± 0.61 μM) (He, 2018). *E*. *microphylla* (extract of leaves with flower buds) showed a significant and highly selective antiproliferative effect on cancer cells (Kudera et al., 2021). At a concentration of 50 μg/mL, the CH extract of *E. microphylla* leaves inhibited MCF-7 and A-549 cancer cell lines by 85.55% and 77.93%, respectively. The DAPI staining method was employed to investigate the mechanism of cell death, which revealed alterations in nuclear morphology in MCF-7 cell lines in the form of distinct changes that were observed during the staining process (Sharma et al., 2022). The extract of the fruit of *E*. *macrophylla* showed dose-dependent antiproliferative activity, possibly influenced by the synergistic and additive effects of individual phenolics (Deng et al., 2020). Both *E*. *tinifolia* and *Sideroxylon lanuginosum* Michaux (Sapotaceae) exhibited antiproliferative activities against HeLa, HT-29 and MCF-7 cells (Monroy-García et al., 2021).

### 4.8 Antihemolytic activity

The antihemolytic properties of the leaf extracts (PE, CH, EA, ethanol, and aqua) of *E. acuminata* were assessed using spectrophotometric technique. The EA extract demonstrated the most potential for antihemolytic activity, with an IC_50_ of 90 μg/mL (Shukla et al., 2021). It was demonstrated that *E. tinifolia* polyphenols had the ability to reduce hemolysis (IC_50_ = 58.55 ± 2.4 μg/mL) (Monroy-García et al., 2021).

### 4.9 Anti-arthritic activity

The methanolic extract of *E. laevis* leaves (500 mg/kg) in mice prevented paw edoema from increasing by 60% and by 56%, as per Velappan and Thangaraj (Velappan and Thangaraj, 2014).

### 4.10 Wound healing activity

With healing times ranging from 7 days to a maximum of 66 days, *E. laevis* leaves show considerable healing properties in both infected and non-infected, chronic and fresh wounds. These properties become more effective as one ages. According to the study, the patient’s immune status remained unaffected because no antibiotics were administered (Thakre et al., 2016). The study also showed that the CH fraction of the EtOH extract of *E. Laevis* has the same wound-healing properties as Povidon iodine ointment in wistar rats (Harne et al., 2021). In 5% and 10% concentrations, the alcoholic extract ointment of *C. retusa* root, stem, and leaves would be able to stimulate wound healing activity. Swiss albino mice were employed to test the effectiveness of wound healing. The ointment prepared from different fraction showed significant effectiveness in wound contraction and faster wound closure compared to the standard Nitrofurazone (0.2%) ointment. As a result, the wound healing study demonstrated that *C. retusa* is available in facilitating wound closure (Mageswari et al., 2012).

### 4.11 Anti-amoebic activity

The leaves of Ampalaya (*Momordica charantia* L.) and Tsaang Gubat *(E. microphylla*) were processed to create a lyophilized aqueous extract. Against Entamoeba histolytica, tsaang gubat and ampalaya leaves did not show any anti-amoebic activity. In reality, they promoted the development of amoebae at all dose levels. The IC_50_ of the extracts of tsaang gubat and ampalaya leaves was greater than 500 μg/mL at 24, 48, and 72 h. These findings contradict the conventional application of these herbal medicines to alleviate diarrhoea (Maramba-Lazarte et al., 2020). The leaves of *E. laevis* were used to create the synthetic silver nanoparticles, which demonstrated larvicidal activity and efficient methylene blue dye degradation. The nanoparticles were also extremely stable in solution and active. After being treated for 72 h, it killed 70% ± 10.24% of *Culex quinquefasciatus* larvae at a dosage of 25 μg/mL. Within 8 h, the nanoparticles reduced Congo red by approximately 85% at a concentration of 200 μg/mL. Furthermore, when exposed to sunlight, the produced nanoparticles may function as a water purifying agent (Panja et al., 2020).

### 4.12 Lipoxygenase inhibitory activity

Compounds **77**, **81**, **84** and **86** isolated from the fresh leaves and twigs of *E. dicksonii* exhibited inhibitory effect against soybean lipoxygenase at a concentration of 10 μg/mL (Dong et al., 2000). Compounds **24**–**27** and **29** demonstrated concentration-dependent inhibition of lipoxygenase, with *K*_i_ values ranging from 0.85 to 57.6 μM. Compounds **26** and **27** were the most potent, having *K*_i_ values of 0.85 μM and 1.0 μM, respectively. Compounds **25**–**27** and **29** exhibited noncompetitive inhibition, whereas compound **24** was classified as an uncompetitive inhibitor of lipoxygenase (Iqbal et al., 2005).

### 4.13 Modulation of gut microbiota

A new polysaccharide called EWMFP was effectively isolated and identified from *E. macrophylla* fruit by Xu group. The impacts on the gastrointestinal microbiota of humans were investigated using an *in vitro* fermentation model that reproduces the micro-ecosystem of the human colon. With a molecular weight of 12.45 KDa, EWMFP is made up of four monosaccharides and is better than inulin at preserving microbial diversity. By the end of fermentation, EWMFP produces a higher concentration of butyrate than inulin and can modify the composition of gut microbes in a different way. Differing routes may lead to differing compositions of short chain fatty acids (SCFA) during fermentation between EWMFP and inulin. According to their research, EWMFP may have a new role as a prebiotic in controlling colonic health (Deng et al., 2020).

### 4.14 Other activities

Erhetianone (**56**), which was isolated from a methanolic preparation of the root bark of *E. buxifolia*, has been shown to have antisnake venom activity against the venom of *Echis carinatus* in rodents. The antisnake venom effect of compound **56** was determined by calculating the LD_50_ of *E. carinatus* venom in mice that was administered subcutaneously (Selvanayagam et al., 1996). It was discovered that the butanolic and EA extracts of *E. philippinensis* bark exhibited anti-histamine releasing activity (Simpol et al., 1994). The objective of this work was to investigate the impact of *E. microphylla* on the quantity of ovarian surface follicles, relative weight of the ovaries and uterus, and folliculogenesis in female Wistar albino rats. Hematology required the removal of the uterus and ovaries. The findings show that 1,000 mg/kg of powdered *E. microphylla* increased the levels of FSH, LH, and estradiol significantly. Additionally, increased folliculogenesis was found along with increased ovarian and uterine weight. As a result, the results point to a noteworthy stimulating effect on female reproductive function that might improve adult female rats’ fertility (Aarthi et al., 2012).

## 5 Toxicology

The alcoholic extract from *E. microphylla* leaves did not exhibit any chromosome-fragmentation generating activity in the mutagenicity or genotoxicity assays (Balboa and Sylianco, 1993; Legaspi and Bagaoisan, 2020). Only one report on acute oral toxicity for *E. laevis* adheres to the OECD recommendation of 423. All of the methanolic extracts from the fruits, stems, and foliage in this investigation were determined to be safe at a dosage of 2,000 mg/kg (Sharma et al., 2021; Velappan and Thangaraj, 2014). The safety and effectiveness of *E. laevis* and *E. microphylla* extracts and fractions for a range of illnesses are not adequately supported by the few and inadequate toxicity reports that are currently available. Moreover, there is a dearth of information in the literature regarding this genus’s toxicity.

## 6 Clinical studies

The tsaang gubat tablets contains a 10% leaf aqueous extract that has been shown to be both safe and efficacious in the treatment of gastrointestinal and biliary colic pain. In a Phase II clinical trial conducted in Pila and Victoria, Laguna, the formulation was administered to five male patients who were diagnosed with acute colic due to lax bowel movements. The following effects were observed after the treatment: (1) all patients reported experiencing relief from intestinal spasms or colic, and (2) the onset of colic relief typically occurred 20–30 min after the dosage. Efficacy, tolerability, and acceptability of the tsaang gubat tablet at a dose of 150 mg/kg were comparable to those of dicycloverine at a dose of 0.5 mg/kg in Phase II clinical trials conducted at the Tarlac Provincial Hospital. The patients were 110 outpatients with acute mild, moderate, or severe biliary colic. The laboratory tests conducted subsequent to the administration of the tsaang gubat tablet did not reveal any abnormalities, and the patients reported no adverse effects (Legaspi and Bagaoisan, 2020). It has been registered with the Philippine Food and Drug Administration, is currently listed in the Philippine National Formulary and has been licensed to a limited number of local pharmaceutical enterprises.

## 7 Cultivation and commercial value

In recent years, there are more and more reports about the cultivation of this genus with the development and utilization of some plants of *Ehretia* genus. It is mainly divided into three cultivation methods. The first one is seed cultivation. The plant fruit begins in ripe and can be collected centrally when the fruit changes from green to orange-red or yellow. The fruit was peeled and stratified after harvest. The seeds are sprouted and sown when the soil thaws in the following spring. The other two methods are root and branch cottage (Huma, 2019; Zhou, et al., 2012). As a kind of Kudingcha, the leaves of *E. thyrsiflora* have great commercial value as a health tea in China (Wang and Huang, 2005). Furthermore, Tupipa (the fruit of *E. macrophylla*) has been developed as a new food in Hengnan County, China, to promote local economic development and help rural revitalization. Up to now, the county planting area of more than 30,000 mus and the sales of Tupipa products reached 100 million yuan in 2023 (http://lyj.hunan.gov.cn/). *E. tinifolia* produces small, globe-shaped yellow drupes measuring up to 8 mm in diameter. These fruits have a sweet taste and have been utilized as both food and medicinal plants in various regions of Mexico and the United States (Monroy-García et al., 2021). *E. microphylla* is considered the most promising species for large-scale production and is used extensively in traditional Philippine medicine. Based on the findings of early experiments, recommendations were made on the cultivation, harvesting, and storage (where applicable) of vegetative propagation (Mageswari and Karpagam, 2015). Some plants of *Ehretia* genus are often used as herb medicine, food, street tree and yard planting, so the cultivation and commercial value of this genus have certain reference value.

## 8 Conclusion and perspectives

The biology, ethnopharmacology, phytochemistry, pharmacology, toxicity, clinical studies, cultivation and commercial value of the *Ehretia* genus were summarized in this paper. This genus is widely distributed across various regions, with some species being particularly diverse. It has been extensively utilized in traditional medicine and food practices. Although there are commonalities in medicinal applications, these vary significantly depending on the species and geographical location. In fact, *Ehretia* species are used to treat skin conditions, pain and inflammation, and digestive issues in many different countries. Numerous pharmacological activities, including the antioxidant, anti-diabetic, and anti-inflammatory characteristics, have been studied *in vivo* and *in vitro* in several species. While most studies have concentrated on crude extracts, certain active compounds have also been evaluated. There is evidence that flavonoids and phenylpropanoids have antioxidant properties.

In Asian countries, ethnic populations employ *E*. *leavis* as masticatories. Undiscovered wound-healing properties have been shown by *E. leavis*. The finest aspect is that it produces a lot of material without requiring the plant to be uprooted since its leaves are effective. Indian scientists have conducted several studies to enhance the germination of multifunctional trees such as *E. leavis*. To learn more about the characteristics of the genus *Ehretia*, molecular studies have also been conducted on a few species. *E*. *tinifolia* has recently been the subject of study that indicates beneficial responses against cardiovascular illnesses, atherosclerosis, and diabetes problems (Panja et al., 2020).

However, many questions remain regarding our comprehensive understanding of the *Ehretia* genus, necessitating further research. First, given their historical applications, certain species warrant more in-depth investigation. Notably, *E. microphylla* is extensively used in China, India, and the Philippines to treat gastrointestinal and biliary colic, diarrhea, spasms, and inflammation. Studies on this species’ antispasmodic activity have revealed its antioxidant, hepatoprotective, antibacterial, anti-inflammatory, antidiabetic, anticancer, and wound-healing properties. These findings may lead to the discovery of novel bioactive compounds. Therefore, additional research should focus on traditionally used plants that have been overlooked but offer clear benefits. Second, despite limited documentation of traditional usage for most species within this genus, abundant resources allow for continued scientific exploration into these species.

Thirdly, despite the fact that extracts from a number of *Ehretia* species have shown strong activity, comparatively few of the chemicals causing these effects have been found in relation to the number of pharmacological investigations that have been carried out. For instance, *E. anacua* is said to exhibit strong anti-diabetic effects *in vivo*; yet, this plant’s secondary compounds have not been found. Therefore, bio-guided isolation should be used to identify the bioactive compounds from these species. Moreover, there is a great chance of finding novel active chemicals in the genus *Ehretia* because of its high degree of endemism. Lastly, although systematic study is still lacking, research on this species focuses mostly on the investigation of its chemicals and biological activity. Establishing quality standards and conducting more complete investigations are vital for future studies to guarantee the integrity and efficacy of the findings pertaining to this genus. It is recommended to add techniques or approaches that can address these gaps in the future research (e.g., metabolomics, high-throughput screening, HPLC-MS). Furthermore, some of the literatures are not high-level papers, and the experimental data can only be used for reference.

In conclusion, further research is required because the phytochemistry and pharmacology of the *Ehretia* genus have not been thoroughly examined. To date, 101 compounds have been identified from this genus, with phenylpropanoids being the most active class. Rosmarinic acid showed different strong activity in this genus. Several species’ medicinal potential has been highlighted by numerous biological investigations, providing a strong basis for further investigation. It is imperative that further research be carried out *in vivo* with suitable dosage levels and controls. To guarantee safety and effectiveness, the side effects connected to the effective doses should also be thoroughly assessed. Given the rich resource base and significant medical value of this genus, continued research is warranted. This paper contributes scientific value to the ongoing development of the genus.
